# Public health interventions to improve the mental health and mental health literacy of young people in resource-limited settings: a systematic review

**DOI:** 10.3389/fpubh.2026.1741504

**Published:** 2026-06-02

**Authors:** Regeru Njoroge Regeru, Katherine Massey, Yan Ran Wee, Jennifer S. Evans, Shirley Sylvester

**Affiliations:** 1 Johnson & Johnson Middle East FZ-LLC, Nairobi, Kenya; 2 Costello Medical Singapore Pte Ltd., Singapore, Singapore; 3 Johnson & Johnson, Zug, Switzerland

**Keywords:** depression, intervention design, mental health, mental health literacy, schizophrenia, systematic review, young people

## Abstract

**Background:**

Young people aged 15–29 experience a substantial mental health burden, with major depressive disorder (MDD) and schizophrenia accounting for as much as half of this burden. Young people living in low- or middle-income countries (LMICs) face challenges to improving their mental health. A substantial treatment gap exists, driven by resource constraints and workforce shortages. Addressing mental health and mental health literacy through public health interventions has the potential to reduce the burden of mental disorders in this population, while meeting the needs of resource-limited settings. Understanding how best to design public health interventions to address both mental health and mental health literacy is key to effectively using resources and maximising benefits.

**Methods:**

Electronic database searches (MEDLINE, Embase, CENTRAL, PsycINFO) were supplemented with non-English database searches (Global Index Medicus, African Journals Online), searches of conference proceedings and other grey literature sources. Eligible studies were conducted in resource-limited settings and reported characteristics of public health interventions. ≥80% of the study population was required to be aged 15–29 years, with MDD and/or schizophrenia.

**Results:**

5,339 records were identified in database searches; 30 studies (26 on MDD and 4 on schizophrenia) were included. Most studies (25 of 30) presented a behaviour therapy intervention, often based on an existing framework (e.g., CBT, behaviour activation). 10 studies incorporated digital health in their programme design, including electronic/computerised versions of behaviour therapy interventions and messaging services to allow participants to contact facilitators for support. Intervention outcomes were assessed using pre-existing validated tools such as Beck Depression Inventory-II (BDI-II) and the 12-item Discrimination and Stigma scale (DISC-12), often adapted to the local context. Mental health literacy outcomes included social support, self-efficacy and cognitive re-appraisal. 25 studies reported statistically significant improvements in mental health outcomes following intervention, compared to baseline and/or usual care. Outcomes were generally assessed over short follow-up durations of ≤6 months.

**Conclusion:**

These findings demonstrate the potential for public health interventions, enabled by digital health, to improve mental health literacy and outcomes in LMICs. Other considerations for programme design included adaptation of pre-existing intervention frameworks and use of validated tools to evaluate outcomes.

**Systematic review registration:**

https://www.crd.york.ac.uk/PROSPERO/view/CRD42024579598

## 1 Introduction

Young people experience a substantial mental health burden. The 2019 Global Burden of Disease study (1) demonstrated that the burden of mental health conditions, measured in disability-adjusted life years (DALYs) and years lived with disability (YLD), increases from age 15 onwards, and is elevated in people aged 15–29 years (2). Globally, across all age groups, 15.6% of YLDs are attributed to mental disorders; however, in young people aged 15–29 years, this proportion is 23.7% for females and 25.6% for males (3).

Among the general population, depression is the second most prevalent mental health condition but accounts for almost 40% of the DALYs associated with mental disorders. While schizophrenia is substantially less prevalent than other mental disorders, it is associated with a disproportionately high burden, accounting for 11.7% of DALYs associated with mental disorders, third behind depression and anxiety (3). A similar pattern is observed in young people aged 15–29 (1, 2).

Over half (62.5%) of all mental disorders manifest before the age of 25, with the median age of onset being 18 years (4), highlighting the importance of addressing mental health among young people as a public health concern. Early detection and intervention for mental disorders has been associated with preventing lifetime disability (5) and changing the trajectory of long-term mental illness (3, 6). In young people, the burden of mental ill health can have a significant impact as such conditions can be debilitating and affect their education (7). Poor mental health is strongly related to other health issues in young people, including substance abuse and poor sexual health (8). Severe mental disorders are also associated with an increased risk of suicide (9). Early intervention for young people with mental disorders therefore has the potential to reduce both YLD and mortality associated with mental ill health.

In 2010, the World Health Organisation (WHO) published a Youth Mental Health declaration, defining tangible and measurable targets for strengthening mental health services for young people in the next 10 years (10). The Youth Mental Health declaration includes public health targets to reduce preventable mortality, improve mental health literacy, recognise young people with mental health needs, improve access to specialist support and increase youth and family participation in service development (10). The WHO has specified priority mental disorders, aligned with the most burdensome conditions in young people, which include depression and schizophrenia (11). In addition, the United Nations (UN) Sustainable Development Goals (SDGs), published in 2015, include goals to promote mental health and prevent mental-ill health (12).

Compounding the burden of mental ill health in young people, those living in resource-limited settings, including low or middle-income countries (LMICs), face additional challenges. About 80% of people living with mental health disorders reside in LMICs (13). While the factors influencing the mental health of people living in LMICs are similar to those in high-income countries (3), studies have demonstrated a substantial treatment gap for these disorders in LMICs (14), partly as a consequence of resource constraints (15).

Improving mental health outcomes in LMICs requires improvements in both service delivery and the availability of the healthcare workforce (16). Currently, LMICs do not have adequate resources, financial and human (17), to meet their populations’ mental health treatment requirements (18). Approximately 74% of LMICs face workforce shortages for treatment of mental health disorders and an estimated 239,000 additional full-time healthcare workers (psychiatrists, nurses and psychosocial care providers) are required to address the current burden of mental health disorders in LMICs (18).

While the availability of mental health services is a key issue, it is important to consider and address other factors which contribute to the low uptake of mental health care among young people. One such factor is mental health literacy, a concept first proposed by Jorm and colleagues in 1997 (19). Under this framework, mental health literacy can be defined as “knowledge and beliefs about mental disorders which aid their recognition, management or prevention”. Within this framework, mental health literacy is comprised of several different components, including: ability to recognise specific disorders; knowledge and beliefs about risk factors and causes; knowledge and beliefs about self-help interventions; knowledge and beliefs about professional help; attitudes which facilitate recognition and appropriate help-seeking; knowledge of how to seek mental health information (20). More recently, the concept of mental health literacy has evolved to incorporate the reduction of stigma related to mental disorders and extend the idea of self-help into help-seeking efficacy more broadly, with help-seeking efficacy meaning “knowing when and where to seek help and developing competencies designed to improve one’s mental health care and self-management capabilities” (21).

Low mental health literacy is associated with mental health conditions such as depression and anxiety (22), as it can contribute to low awareness of mental disorders and mental health services, as well as sociocultural barriers to seeking help such as stigma (13). Accordingly, previous studies show that health literacy interventions, including cognitive behavioural therapy, psychoeducation and coaching, have a positive impact on reducing the symptoms of mental health conditions such as depression and anxiety (23). In schizophrenia, poor health literacy can contribute to poor health management and the risk of relapse through patients’ lack of awareness of their illness and lack of understanding of the importance of treatment (24, 25). Mental health literacy interventions such as psychoeducation are recognised as an important component of managing the condition (26, 27). Improving mental health literacy is therefore a key aspect to improving the mental health of young people, including those living with mental disorders.

Mental health and mental health literacy can be addressed by public health interventions with a focus on preventing mental health disorders, promoting mental wellbeing and promoting resilience to support recovery or remission from mental disorders (28). Public health interventions can help to address mental health-related social stigma through implementation of family or community-based support (such as school- or university-based mental health programmes) to extend existing support networks for people with mental health disorders (29). In addition, it has been suggested that the mental health gap in LMICs can instead be closed through the innovative use of existing human resource, rather than reliance on scarce expert healthcare providers (30). Task shifting public health interventions to address mental health concerns have previously been demonstrated to be successful and cost-effective (31, 32). Interventions to address mental health literacy can thus include those aiming to increase awareness and education (proximal outcomes) as well as those improving access to care such as behaviour therapy and counselling though self-help, telemedicine or peer-to-peer programmes, which could more directly target distal outcomes (improving mental health functioning) (33).

While public health interventions have potential to improve the mental health and mental health literacy of young people, particularly those already affected by mental disorders, it is important to ensure that such intervention programmes are appropriately designed and effective. Given the high morbidity in young people, this is of particular concern in resource-limited settings, where there is already substantial pressure on mental health services (17, 18). We conducted a systematic literature review (SLR) to identify the characteristics of successful public health interventions in improving the mental health and mental health literacy of young people with serious mental illness (major depressive disorder [MDD] or schizophrenia) in resource-limited settings.

## 2 Methods

The protocol for this SLR was registered on the International Prospective Register of Systematic Reviews (PROSPERO; CRD42024579598) and is reported according to the Preferred Reporting Items for Systematic Reviews and Meta-Analysis (PRISMA) 2020 guidelines (34). All amendments to the information provided at protocol registration are detailed in the PROSPERO record.

### 2.1 Eligibility criteria

Both interventional studies (randomised controlled trials [RCTs] and non-randomised interventional studies) and observational studies were eligible if they reported a public health intervention. Studies must have been carried out in a resource-limited setting, including in any country not classified by the World Bank as high income (35), or in an economically disadvantaged population in a high-income country. At least 80% of the participants included in the study were required to be young people aged 15–29 years with MDD and/or schizophrenia. This criterion was applied given the non-standard age range of interest to this review, ensuring that studies which included a majority of participants of interest would not be missed. Studies must have reported the characteristics of public health interventions, characteristics of the target population, and qualitative or quantitative assessments of the intervention outcome. Outcomes could be reported at any time point or after any period of follow up. There were no restrictions on the language of the publication. Case reports, studies focusing on populations of children or older adults and studies primarily investigating pharmacological interventions were excluded. Full inclusion and exclusion criteria for the SLR are provided in the Supplementary Table 7.

### 2.2 Information sources and search strategy

Searches were conducted in July 2024 in the following electronic databases: MEDLINE (including MEDLINE In-Process, MEDLINE Daily and MEDLINE Epub Ahead of Print), Embase, The Cochrane Central Register of Controlled Trials (CENTRAL) and PsycINFO. MEDLINE, MEDLINE In-Process, MEDLINE Epub Ahead of Print, Embase and CENTRAL were searched simultaneously via the Ovid SP platform. PsycINFO was searched via the APA PsychNet platform.

Additional searches were conducted in the following non-English databases: Global Index Medicus (including African Index Medicus, Index Medicus for the Eastern Mediterranean Region, Index Medicus for the South-East Asia Region, Latin America and the Caribbean Literature on Health Sciences and Western Pacific Region Index Medicus) and African Journals Online.

The bibliographies of SLRs or network meta-analyses identified in the electronic database searches that met the eligibility criteria were hand-searched to identify further relevant studies. Conference proceedings (European Congress of Psychiatry, Karolinska Institutet – UNICEF Joint Conference on Global Child and Adolescent Mental Health, European Society for Child and Adolescent Psychiatry and International Association for Child and Adolescent Psychiatry and Allied Professionals) from July 2021–June 2024 were searched to identify relevant congress abstracts. Conference searches were restricted to the three years prior to the start of the review, under the assumption that high quality conference presentations would subsequently be published as journal articles and captured via database searches. The WHO website and websites of non-governmental organisations that focused on mental health and young people (Being Initiative, Global Coalition for Youth Mental Health, Orygen and Verywell Mind) were also searched. Search strategies for each information source are reported in the Supplementary Data.

### 2.3 Data collection and extraction

Abstracts were reviewed within a bespoke literature review platform. Two independent reviewers screened the title and abstract of each record (stage 1), as well as the full-texts of all potentially eligible records identified in stage 1 (stage 2). A third reviewer resolved any disagreements. Decisions and reasons for inclusion or exclusion at each stage were recorded.

Data extraction was performed by a single individual for each study. Once the initial extraction was completed, a second individual verified all extracted information. Any discrepancies or missing information was discussed by both individuals until a consensus was reached. If necessary, a third reviewer made the final decision.

Data were extracted into a pre-specified extraction grid in Microsoft Excel. Where possible, effect measures were extracted from included studies. Information which was not provided in the individual studies was noted as “not reported” in the extraction grid. Items which were extracted included study characteristics, intervention characteristics, characteristics of the target population and intervention outcome assessment. Full details of extracted items are provided in the Supplementary Data.

Due to the diverse nature of the outcomes of interest, which precluded meta-analyses, a narrative synthesis was conducted. All studies meeting the review eligibility criteria were included in the narrative synthesis. The characteristics of the included studies were assessed first, followed by classification of programmes based on the type of public health intervention assessed and the setting. Study populations were stratified by diagnosis (MDD or schizophrenia) and the assessment of outcomes was stratified by the perspective of each outcome (mental health care recipient perspective and healthcare system perspective). Author perspectives on the replicability and scalability of public health interventions were also considered.

### 2.4 Assessment of risk of bias in included studies

The quality of all included studies was assessed using the Alberta Heritage Foundation for Medical Research (AHFMR) tool for evaluating primary research papers from a variety of fields (36). The AHFMR tool assesses whether quantitative studies have an appropriate design, clear and suitable participant selection, reliable outcome measures, and adequate control of confounding factors. Quality assessments were performed by a single individual for each included study. A second individual verified the quality assessment. Any discrepancies were discussed by both individuals until a consensus was reached. If necessary, a third individual made the final decision.

Studies were scored on a scale of 0.00 to 1.00, where 1.00 signified the highest quality study scoring the best rating of “Yes” for every question in the quality assessment.

## 3 Results

**Figure 1:**
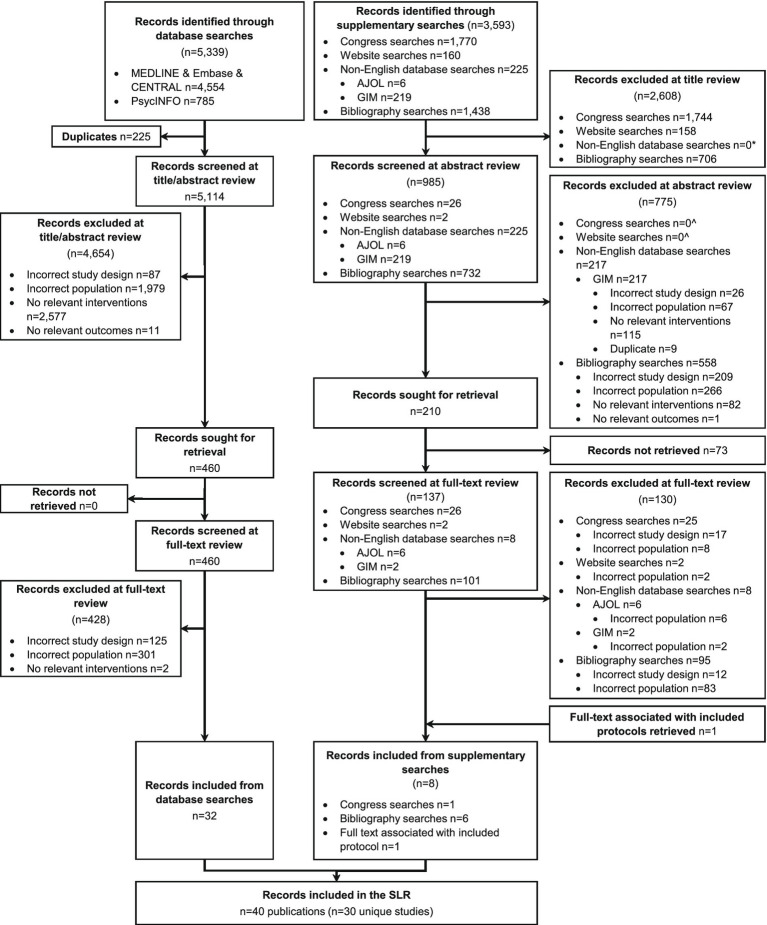
PRISMA flowchart. *Records identified through the non-English databases were not reviewed at title stage and were brought directly to abstract review stage. ^After title review stage, records identified through congress and website searches were not reviewed at abstract stage and were brought directly to full-text review stage. AJOL, African Journals Online; CENTRAL, Cochrane Register of Controlled Trials; GIM, Global Index Medicus; SLR, systematic literature review.

This SLR was conducted to identify the characteristics of successful mental health literacy interventions for young people with MDD or schizophrenia in resource-limited settings. The PRISMA flowchart is presented in Figure 1. A total of 5,339 publications were retrieved from the electronic database searches. After title/abstract screening, 460 potentially relevant records were selected for full-text review, of which 32 were included. Supplementary searches yielded 3,593 records, of which seven met the inclusion criteria. In total, this SLR identified 40 publications reporting on 30 unique studies. This included one study for which only a published protocol was identified (37).

The quality of all included studies was assessed using the AHFMR checklist (36). Study quality was heterogenous; four studies received a full score of 1.00 by obtaining a positive assessment in every applicable category. Among the remaining studies, quality assessment scores ranged from 0.61 to 0.96. The majority of studies (27 of 30) scored >0.75 in the quality assessment, indicating that the evidence base identified in this review is overall of good quality, supporting the conclusions drawn from the evidence. Full results of the quality assessment are included in the Supplementary Data.

All studies clearly described research objectives and used study designs appropriate to the research question, and most also appropriately described the outcome/exposure measures and analytic methods (93% of studies for both). Where applicable (N/A for one study protocol with no results available), the majority reported participant characteristics (90% of studies) and study results (90% of studies) in sufficient detail. The checklist items on which a higher number of studies scored poorly were the use of random allocation, investigator blinding and participant blinding (where this would have been possible).

Two studies of interventions in young people with MDD scored ≤0.75 on the quality assessment, meaning outcomes from these studies should be interpreted with more caution. Both trials were RCTs investigating an electronic cognitive behaviour therapy (CBT) intervention which measured outcomes using pre-existing validated scales (Beck Depression Inventory-II [BDI-II] and Patient Health Questionnaire-9 [PHQ-9]) (38, 39). However, both studies did not fully describe the random allocation of participants, or the blinding of investigators and participants. Issues were also identified with the level of detail in the reporting of results. One further study in young people with schizophrenia was only available as a conference abstract, with limited information reported (40); as such, limited information from this study was considered as part of the descriptive synthesis.

### 3.1 Study characteristics

Of the 30 studies, 29 were interventional in design (37–39, 41–66), of which 25 studies were RCTs (37–39, 42, 43, 45–53, 56–66). Eighteen studies were conducted across multiple centres (37, 39–43, 45, 47, 50–55, 58, 62, 64, 65). A summary of study characteristics is presented in Table 1.

**Table 1:** Study characteristics.

Study name	Geography	Study design	Funding and development partners
MDD			
Alavi 2013 (41)	Iran	Interventional non-RCT, multicentre	Funding: Shiraz University of Medical Sciences Development partners: Namazi, Shooshtari, and Hafez Hospitals
Alhusen 2021 (42)	United States	RCT, multicentre	NR
Arjadi 2018 (43)	Indonesia	RCT, multicentre	Funding: Indonesia Endowment Fund for Education, Ministry of Health, Republic of Indonesia; University of Groningen Development partners: Utrecht University
Bantjes 2021 (44)	South Africa	Interventional non-RCT, single centre	Funding: South African Medical Research Council; Division of Research Development at Stellenbosch University
Benjet 2023 (45)	Mexico; Colombia	RCT, multicentre	Funding: National Institute of Mental Health; Fogarty International Centre Development partners: Universidad Cooperative de Colombia; la Red de Universidades la Salle; Universidad Autónoma Metropolitana; Universidad National Autónoma de México
Chen 2024 (38)	China	RCT	Funding: CXTQ Foundation Development partners: Li Chiu Kong Family Sleep Assessment Unit, Department of Psychiatry, The Chinese University of Hong Kong; BestCare & SuMian BioTech
Church 2012 (46)	Philippines	RCT, single centre	NR
Eseadi 2022 (47)	Nigeria	RCT, multicentre	Development partner: University of Nigeria
Ezeudu 2020 (48)	Nigeria	RCT, single centre	Development partner: University of Nigeria
Far 2017 (49)	Iran	RCT, single centre	Development partner: Tehran University
Fereydouni 2022 (50)	Iran	RCT, multicentre	Funding: Projekt DEAL Development partner: Gachsaran University; Yasuj University; University of Siegen
Gureje 2019 (52)	Nigeria	RCT, multicentre	Funding: Grand Challenges Canada; College of Medicine, University of Ibadan
Gureje 2022 (51)	Nigeria	RCT, multicentre	Funding: Global Affairs Canada; Canadian Institutes of Health Research; Canada’s International Development Research Centre
Kaaya 2022 (53)	Tanzania	RCT, multicentre	Funding: National Institute of Mental Health
Kondradt 2018 (54)	Brazil	Interventional non-RCT, multicentre	Funding: Políticas Públicas para o SUS programme, of Conselho Nacional de Desenvolvimento Científico e Tecnológico
Nakku 2021 (55)	Uganda	Interventional non-RCT, multicentre	Funding: UK Government aid Development partners: PRIME; UWONET
Nejati 2019 (56)	Iran	RCT, single centre	Funding: Iranian MSRT, Deputy of Scholarship and Students Affairs
Ofoegbu 2020 (39)	Nigeria	RCT, multicentre	NR
Olashore 2023 (58)	Botswana	RCT, multicentre	Funding: University of Botswana
Osborn 2020 (59)	Kenya	RCT, single centre	Funding: Shamiri Institute
Savari 2021 (60)	Iran	RCT, single centre	Development partner: Bu-Ali Sina University
Singla 2021 (62)	India/Pakistan	RCT, multicentre	Funding: National Institute of Mental Health Development partner: South Asian Hub for Advocacy, Research and Education on Mental Health
Srivastava 2020 (63)	India	RCT, single centre	Funding: Indian Council of Medical Research
Toth 2013 (64)	United States	RCT, multicentre	Funding: National Institute of Mental Health
Xu 2023 (65)	China	RCT, multicentre	Funding: National Natural Science Foundation of China; General Scientific Research Project of Shanghai Municipal Health Commission Development partner: Kunming Medical University
Zemestani 2020 (66)	Iran	RCT, single centre	Development partner: State Welfare Organisation (Tooska Institute)
Schizophrenia			
Correa-Oliveira 2022 (40)	Brazil	Retrospective observational multicentre study	NR
Mlay 2022 (37)	South Africa	RCT, multicentre	Funding: Swedish International Development Agency; UK Global Challenge Research fund; KRISP with a core award from the Technology Innovation Agency of the Department for Science and Technology; Development partner: University of KwaZulu-Natal
Ngoc 2016 (57)	Vietnam	RCT, single centre	Funding: US NIH Fogarty International Centre; NIH Office of the Director; National Institute of Mental Health
She 2016 (61)	China	RCT, single centre	Development partners: Wuhan University; HOPE School of Nursing; Wuhan Mental Health Centre

KRISP, KwaZulu-Natal Research Innovation and Sequencing Platform; MDD, major depressive disorder; MSRT, Ministry for Science, Research and Technology; NIH, National Institutes of Health; NR, not reported; PRIME, Programme for Improving Mental Health Care; RCT, randomised controlled trial; UK, United Kingdom; US, United States; UWONET, Uganda Women’s Network.

The majority of studies were conducted in the Asia-Pacific region (14 studies) (38, 41, 43, 46, 49, 50, 56, 57, 60–63, 65, 66) and Africa (11 studies) (37, 39, 44, 47, 48, 51–53, 55, 58, 59). Two of the included studies reported on interventions implemented in resource-limited settings in North America. Resource-limited settings were identified as targeting participants of low socioeconomic status; one of the two studies specified that participants had to reside at or below the United States (US) federal poverty level to be eligible (42, 64). In total, 17 countries were represented across the 30 studies.

Over half of the studies (16 of 30) listed a university as a source of funding or a key development partner (37, 38, 41, 43–45, 47–50, 52, 58–61, 65). Other partners and funders included governmental bodies (11 studies), such as National Institutes of Health (43, 45, 51, 53–57, 62–65). Five studies were supported by funding from the US National Institute of Mental Health (45, 53, 57, 62, 64).

### 3.2 Programme characteristics

The SLR identified 26 studies with a focus on MDD (38, 39, 41–56, 58–60, 62–66) and four studies with a focus on schizophrenia (37, 40, 57, 61). The characteristics of the programmes reported in these studies are presented in Table 2.

**Table 2:** Programme characteristics.

Study name	*N*	Mental health criteria	Other eligibility criteria	Study setting	Intervention category	Intervention(s)	Intervention duration and frequency	Comparator(s)
MDD								
Alavi 2013 (41)	30	Mild to moderate MDD, assessed using BDI	Patients with suicidal attempts in the past 90 days, with mild to moderate MDD	Specialist/hospital; Namazi, Shooshtari, and Hafez Hospitals, Iran	Behaviour therapy	CBT, with components including psychoeducation, developing reasons for living and hope, skills training (including behavioural activation, problem solving, mobilising social support and assertiveness skills) and relapse prevention	12 sessions over 3 months	Waitlist-controlled; intervention commenced 3 months after enrolment
Alhusen 2021 (42)	60	Moderate to severe depressive symptomatology (EPDS score >12)	Women aged ≥16 years; <12 weeks gestation at the time of enrolment	Maternal/child care centre; 2 obstetrical clinics, Baltimore, Maryland, US	Behaviour therapy	CBT-based Mothers and Babies Course, focusing on helping participants manage their daily experiences and challenges. Delivered in a group setting by trained facilitators (clinical social worker and family nurse practitioner)	2-h intervention sessions conducted weekly over 6 weeks	Usual prenatal care
Arjadi 2018 (43)	313	Depressive disorder, scoring ≥10 on PHQ-9; met the criteria for MDD or persistent depressive disorder based on structured clinical interview for DSM-V	Aged ≥16 years; could use the internet	Community-based (mixed urban and rural setting)	Behaviour therapy; Telemedicine	Behavioural activation (GAF-ID), internet-based intervention for depression guided by trained lay counsellors and adapted for the Indonesian context	8 weekly structured modules to be completed in 30–45 min each	Minimal psychoeducation, delivered online
Bantjes 2021 (44)	158	Moderate or severe symptoms of depression assessed, using PHQ-9	Students who completed a baseline assessment and provided informed consent	University/school; Stellenbosch University, South Africa (urban setting)	Behaviour therapy; Telemedicine	Group CBT intervention, conducted over Microsoft Teams and facilitated by registered counsellors and clinical psychology master’s students under the supervision of a registered psychologist. Participants were provided with interactive PDF workbooks consisting of exercises and brief summaries of the main ideas and skills for each session before each workshop	10 workshops of 60–75 min duration, conducted weekly	None
Benjet 2023 (45)	1,319	Moderate, moderately severe or severe depression, scoring ≥10 on PHQ-9	NR	University/school; 7 universities in Colombia and Mexico	Behaviour therapy; Telemedicine	Guided or unguided internet-based CBT included behavioural activation and cognitive restructuring delivered via texts, testimonials, audio, videos, quizzes, exercises, and homework. CBT programme was culturally adapted to the setting. In the guided version, supporters (bachelor-level graduates of psychology or behavioural health programmes) provided feedback and personalised recommendations (e.g., revisiting modules), while in the unguided version, users accessed the platform without tailored support.	8 modules with users recommended to complete 1 per week; users able to access the programme for up to 12 months	Usual care (including medication, psychotherapy or referral to a community treatment provider)
Chen 2024 (38)	113	MDD and comorbid insomnia, assessed by clinical interview	Chinese young people aged 15–25	NR	Behaviour therapy; Health education and patient education; Telemedicine	Electronic-based CBT for insomnia	6 weeks	Electronic-based health education
Church 2012 (46)	18	Moderate or severe depression, scoring ≥19 on BDI	First-year university students	University/school; University of Santo Tomas, Manila	Behaviour therapy	EFT, group treatment combining exposure, cognitive reprocessing, and somatic stimulation. Delivered by a trained student who served as group facilitator.	Four 90-min sessions administered within 3 consecutive weeks	None
Eseadi 2022 (47)	67	Moderate to severe depression, scoring ≥20 on BDI-II	First-year undergraduate religious education; not involved in other depression treatment programmes	University/school; 4 universities in southern Nigeria	Behaviour therapy	RREBT, faith-based intervention that incorporates scriptural contents and other religious resources relevant to an individual’s religious traditions and orientates individuals to the treatment process for resolving their emotional and/or behavioural problems. Adapted from standard REBT by incorporating religious aspects	One 2-h session per week for 12 weeks	Waitlist-controlled; intervention for control group participants commenced 1 week after study follow-up evaluation
Ezeudu 2020 (48)	23	MDD, scoring ≥31 on BDI-II	Undergraduate students	University/school; University of Nigeria, Nsukka	Behaviour therapy	REBT, using cognitive, behavioural, and emotive techniques to assist patients in identifying and altering their depressive beliefs, seeks to modify irrational and self-defeating thoughts and beliefs	12 weeks	Usual care (counselling)
Far 2017 (49)	19	MDD diagnosis using structured clinical interview for DSM-IV	Diagnosed with MDD by structured clinical interview for DSM-IV; received no psychological therapies during 6 months before participation in the study; aged between 18 and 35 years	University/school; Tehran University, Iran	Behaviour therapy	ACT, with content including identifying experiential avoidances, cognitive defusion, acceptance, mindfulness exercise and clarifying values	12 sessions in total, administered twice per week	CT
Fereydouni 2022 (50)	60	Elevated depression symptoms, scoring ≥22 on BDI-II	Students at a participating university	University/school; Islamic Azad University, Iran	Health education and patient education; Self care	Spiritually sensitive logotherapy, administered according to a manual by a trained psychotherapist qualified in CBT and logotherapy, Logotherapy was adapted by the authors in include religious values and spiritual practises	12 weekly group sessions of 120 min duration	Supportive group discussions that did not address the topics covered in the logotherapy group
Gureje 2019 (52)	686	EPDS score ≥12 and confirmed DSM-IV diagnosis of depression	Women aged 16–45 years; gestational age between 16 and 28 weeks	Maternal/child care centre; 137 maternal and child care clinics in Oyo State, Nigeria (mixed rural and urban setting)	Behaviour therapy; Collaborative care; Health education and patient education; Health services; Telemedicine; Patient care planning and management	PST for Primary Care was delivered by maternal care providers, addressing pregnancy, childbirth, marital relationships, and parental roles. It incorporated the treatment specifications of the WHO mhGAP-IG adapted for Nigeria. Support and consultation were via mobile phones, except when face-to-face assessment was indicated. Mothers received automated reminders for appointments and therapy-related tasks through voice messages and calls.	30–45-min sessions. In the antenatal period, 8 sessions delivered weekly. Starting at 6 weeks postpartum, session number and frequency was dependent on EPDS score: 4 fortnightly sessions (EPDS <12); 8 weekly sessions (EPDS ≥12)	Enhanced usual care with standard treatment for depression based on WHO mhGAP-IG
Gureje 2022 (51)	242	EPDS score ≥12 on EPDS to confirm presence of major depression according to DSM-IV criteria	Aged <20 years; absence of psychotic symptoms and bipolar disorder; foetal gestational age <36 weeks	Maternal/child care centre; Primary maternal care clinics, Ibadan, Oyo State, Nigeria	Behaviour therapy; Collaborative care; Health education and patient education; Telemedicine	Intervention package consisting of behavioural activation and PST, parenting skills training, and social and parenting skills support, delivered by the maternal care providers. Adolescents addressed psychosocial stressors through guided problem-solving. Parenting skills training was delivered during face-to-face sessions as a component of problem-solving treatment, and through mobile phone calls and texts. Social and parenting skills support was provided by a “neighbourhood mother,” an experienced older woman. The maternal care provider made regular phone contacts with the neighbourhood mother to exchange updates on the adolescent mother’s progress.	6 sessions in the antenatal period, with frequency dependent on EPDS score: 3 weekly sessions followed by 3 fortnightly sessions (EPDS score 12–17); 6 weekly sessions (EPDS score >17)	Usual care (psychoeducation and addressing current psychosocial stressors)
Kaaya 2022 (53)	742	Depressive symptoms comparable with MDD, scoring ≥9 on PHQ-9	≥18 years; gestational age <30 weeks; HIV-infected and receiving ART; had no current plan to harm themselves; planned to continue postpartum care at the study facility	Outpatient/primary care; Government-managed health centres	Behaviour therapy; Collaborative care; Patient care planning and management	PST and CBT, stepped-care model with evidence-based components from PST and CBT implemented by trained lay facilitators.	During pregnancy: 6 group therapy sessions and 1 -pre-delivery orientation session. Postpartum: 8 once-weekly small group sessions	Enhanced usual care
Kondradt 2018 (54)	91	Clinical diagnosis of MDD according to the Portuguese version of the structured clinical interview for DSM-V	Young adult aged 18–29 years	Community-based; Community health centre, centres of psychosocial assistance, and the local media	Behaviour therapy	Brief CT (CBT and NCT)	7 sessions	None
Nakku 2021 (55)	153	Screened for probable depression using PHQ-9 (score of ≥5), confirmatory diagnosis performed using WHO mhGAP-IG algorithm	In the second or third trimester of pregnancy; residing in the area of study; aged ≥18 years; spoke the study languages (English or Luganda)	Outpatient/primary care; Public health facilities in Kamuli district, Uganda (rural setting)	Behaviour therapy; Collaborative care; Health education and patient education	GPST, midwife-led intervention conducted as part of a mental health care plan	Minimum of 4 monthly sessions, lasting for 1.5–2 h, coinciding with scheduled antenatal visits. Women could return for more sessions if they wished	None
Nejati 2019 (56)	22	Moderate to severe depression using depression scale of DASS-42; moderate to severe depression, scoring ≥20 on BDI-II	Aged 18–21 years; scoring below the average in anxiety scale on the DASS-42; not on antidepressants or other psychotropic medications; not receiving any psychological intervention	University/school; Mental health clinic, Shahid Beheshti University, Iran	Behaviour therapy; Telemedicine	AHP, cognitive training programme for modifying negative interpretation and attention biases	10 sessions conducted twice per week over 5 weeks	Control tasks that were identical to the AHP condition except that no feedback was given and the targets appeared mostly in neutral way
Ofoegbu 2020 (39)	114	Depression (screening criteria not described further)	Not currently on any depression intervention or psychotherapy; have a laptop, tablet or smartphone that accesses the internet	University/school; Nigerian federal universities	Behaviour therapy; Telemedicine; Self care	ICBT, involved online materials, an initial telephone support session, and regular guidance from the therapists via phone and internet. It covered psychoeducation, interactive peer support, cognitive disputation, assignments, role play, and depression management. Participants worked independently with twice-a-week guidance from therapists within the portals and through email and phone. They could also seek additional support as needed.	10 weekly sessions	Usual care
Olashore 2023 (58)	50	Met the criteria for depression as indicated by MINI-KID (score of 9–14 on PHQ-9)	Aged 15–19 years; living with HIV; on ART for at least 6 months; not on psychotropic drugs; poorly adherent to ART	Maternal/child care centre; Medical clinics at Botswana Baylor Children’s Clinical Centre of Excellence	Behaviour therapy; Health education and patient education	Psychological interventions including psychoeducation, PST, and rehearsal strategies, sessions were delivered by a trained graduate psychology counsellor. Intervention involved interactive discussion, roleplay, and brief 10-min plenary sessions in the local language, mixed with English	Five 1-h sessions conducted weekly	Usual care
Osborn 2020 (59)	103	Elevated depressive symptoms, scoring ≥10 on PHQ-8	Students aged 13–18	University/school; Mixed-gender private boarding secondary school, Kiambu County, Kenya	Self care; Telemedicine	Shamiri (group-based programme), digital self-help version of Shamiri, a 3-component intervention that has previously been delivered in person, in groups, over 4 weeks with content that included growth mindset, gratitude, and value affirmation	One 1-h session	Study skills activities
Savari 2021 (60)	30	Diagnosis of MDD confirmed by structured clinical interview for DSM-V and scoring ≥20 on BDI-II	Aged 20–30	University/school; Bu-Ali Sina University, Iran	Behaviour therapy	CMT, intervention provided in group sessions by trained MSc students, addressing themes including compassion, mindfulness, and managing self-criticism	Eight 90-min sessions twice a week over 4 weeks	Usual care
Singla 2021 (62)	850	Moderate to severe depressive symptoms, scoring ≥10 on PHQ-9	Pregnant women in second or third trimester	Maternal/child care centre; Participant homes, antenatal clinicals, primary health centres in Goa, India; community settings, rural subdistrict of Punjab, Pakistan (mixed urban and rural setting)	Behaviour therapy; Collaborative care; Health services; Self care	Behaviour activation through the Think Healthy Programme, delivered by peers (women with children, a similar sociodemographic background and good communication skills). It used simplified cognitive and behavioural elements, employing active listening, family collaboration, guided discovery, homework, and behavioural activation to replace unhealthy behaviours with healthy ones.	Depending on the trimester of recruitment (second or third), 6–14 sessions of 30–45 min over 7–12 months	Enhanced usual care (informing participants of their diagnosis of depression, providing information on how and where to seek healthcare from)
Srivastava 2020 (63)	21	Diagnosis of mild to moderate unipolar depression using ICD-10	Adolescents aged 13–19 years; working knowledge of computers; medication stabilised for 4 weeks	Specialist/hospital; Outpatient clinic of a tertiary care hospital, India (mixed urban and rural setting)	Behaviour therapy; Telemedicine	“Smart Teen,” a computer assisted CBT, web application consisting of 12 sessions of CBT. Platform included practise items, case vignettes, personal diaries and a tool kit (where learnt skills are added with a shortcut link) in addition to the sessions. Smart Teen was developed to augment standard in-person CBT and participants continued to receive pharmacotherapy from a psychiatrist.	12 weekly sessions	Usual care (pharmacotherapy and 12 weekly sessions of active psychological therapy)
Toth 2013 (64)	128	Diagnosis of MDD using DSM-IV; scoring ≥19 on BDI-II	Non-treatment seeking women aged 18–40 with a 12-month-old infant; residing at or below US federal poverty level	Community-based; Clinical or home settings (urban setting)	Behaviour Therapy; Health education and patient education; Health Services; Self Care	IPT, addressing interpersonal aspects of depression, emphasising problem-solving and social support within a relational therapy model. The intervention was conducted by experienced therapists.	One 1-h session per week for 14 weeks	Usual care
Xu 2023 (65)	48	Diagnosis of MDD using DSM-V	Undergraduate students	University/school; Universities in Kunming, Yunnan, China	Behaviour therapy	MBCT, group intervention, mindfulness and cognitive skills taught included being aware of and recognising one’s own bodily sensations, attending to distressing thoughts and feelings, and cultivating acceptance and self-awareness. Homework consisted of a protocol for measuring/observing negative feelings, body–mind awareness exercises, a day-to-day motivated chart, etc.	Eight 2.5-h weekly sessions	8 weekly 2.5-h mental health care sessions for depression, anxiety, and general psychological problems (including psycho-education, self-confidence training, interpersonal skills training and general support group sessions) provided by a university psychology counsellor
Zemestani 2020 (66)	60	Met criteria for moderate or severe depression on DSM-V; scoring ≥20 on BDI-II	Meet the WHO-ICF criteria for moderate impairment in physical disability; have at least a high school diploma	Outpatient/primary care; Clinical rehabilitation centre of State Welfare Organisation (Tooska Institute) in Kamyaran, Kurdistan, Iran	Behaviour therapy; Health education and patient education; Self care	ACT, didactic presentations and group mindfulness exercises aimed at developing cognitive flexibility, delivered by a trained clinical psychologist	Eight 90-min group sessions, 1 per week for 8 weeks	Usual care (psychoeducation for depression, once per week for 8 weeks)
Schizophrenia								
Correa-Oliveira 2022 (40)	237	People with schizophrenia	NR	Outpatient/primary care; Outpatient service in the Ribeirão Preto catchment area, Southeastern Brazil	Health services	Ribeirão Preto Early Intervention for Psychosis Programme	NR	None
Mlay 2022 (37)	NR	People with first episode psychosis	Aged 18–29 years; on antipsychotic medication for <6 months after initial psychiatric hospital visit; resident in catchment area for ≥6 months; unemployed	Specialist/hospital; Specialised government psychiatric facilities located in KwaZulu Natal, South Africa	Patient care planning and management	Enhanced usual care that included refilling antipsychotic medication, planning for the next visit, counselling services and constant reminder by SMS or call twice in the last week before a visit. This was supplemented with an unconditional cash transfer	Monthly unconditional cash transfer provided for 3 months	Enhanced usual care, without the cash transfer
Ngoc 2016 (57)	59	ICD-10 diagnosis of schizophrenia	≤3 prior psychiatric hospitalisations including the current one; a duration of <3 years for their schizophrenia; age 18–30 years; family living within 50 kilometres of the hospital	Specialist/hospital; Danang Psychiatric Hospital, Vietnam	Health education and patient education	Family schizophrenia education programme, targeting family members as well as the patient. Interactive sessions conducted by a hospital psychiatrist, 2 psychologists and 2 nurses	Three 1.5-h sessions conducted over 1.5 weeks	Usual care
She 2016 (61)	60	DSM-IV diagnosis of schizophrenia	In the recovery stage with a PANSS score ≤80; aged 16–18 years; middle school education background or higher; able to complete the outcome assessment scales independently	Specialist/hospital; inpatient adolescent unit of a major mental health centre in central China (urban setting)	Behaviour therapy; Self care	Behaviour programme (group therapy activity intervention), conducted by a psychotherapist and a psychiatric nurse. Participants also received usual inpatient care	Twelve 1-h sessions held twice per week for 6 weeks	Usual inpatient care with 12 sessions of handicraft activities

ACT, acceptance and commitment therapy; AHP, Ambiguous Hallmark Programme; ART, antiretroviral therapy; BDI, Beck Depression Inventory; CBT, cognitive behaviour therapy; CMT, compassionate mind training; CT, cognitive therapy; DASS, Depression, Anxiety and Stress Scale; DSM, Diagnostic and Statistical Manual of Mental Disorders; EFT, emotional freedom techniques; EPDS, Edinburgh Postnatal Depression Scale; GAF-ID, Guided Act and Feel-Indonesia; GPST, group problem solving therapy; HIV, human immunodeficiency virus; ICBT, internet-based CBT; ICD, International Classification of Diseases; IPT, interpersonal psychotherapy; MBCT, mindfulness-based cognitive therapy; MDD, major depressive disorder; mhGAP-IG, mental health gap action plan-intervention guide; MINI-KID, Mini International Neuropsychiatric Interview for Children and Adolescents; NCT, narrative cognitive therapy; NR, not reported; PANSS, Positive and Negative Syndrome Scale; PHQ, Patient Health Questionnaire; PST, problem solving therapy; (R)REBT, (religious) rational emotive behaviour therapy; SMS, short messaging service; WHO, World Health Organisation.

Studies of MDD most commonly used BDI-II (7 studies) (47, 48, 50, 56, 60, 64, 66) and PHQ-9 (7 studies) (43–45, 53, 55, 58, 62) to assess individuals for study eligibility. One study conducted in a Kenyan school setting used an adaptation of the PHQ-9 that excluded the suicidal ideation item, as prior research conducted in this setting indicated that this item could be stigmatising and alienating to students (59).

Some studies using PHQ-9 and BDI-II reported pre-specified score thresholds to identify individuals with symptoms consistent with MDD; however, there was variation in the thresholds used between studies. For BDI-II, a threshold of 19–22 was most commonly used (47, 50, 56, 60, 64, 66), though one study specified that participants were required to have a score ≥31 (48). For PHQ-9, five studies used a score of ≥9 or ≥10 to identify MDD (43, 45, 53, 58, 62) while one study screened participants for probable depression using PHQ-9 and a cut-off of ≥5, before confirmatory diagnosis using the WHO Mental Health Gap Action Plan Intervention Guide (mhGAP-IG) algorithm (55). Additionally, a small number of studies used both a validated scale in combination with a version of the Diagnostic and Statistical Manual of Mental Disorders (DSM) to confirm a diagnosis of depression (43, 49, 51, 52, 60, 64, 66).

Seven studies were specifically interested in perinatal depression or mothers with MDD (42, 51–53, 55, 62, 64). Among these, three used the Edinburgh Postnatal Depression Scale (EPDS) to identify participants with perinatal depression (42, 51, 52). The remaining four studies used PHQ-9 (53, 55, 62) and BDI-II (64) to identify MDD. In three studies, confirmation of perinatal depression was performed using an additional diagnostic tool: DSM-IV (52, 64) or the WHO mhGAP-IG algorithm (55).

Among the four studies which recruited individuals with schizophrenia, two used disease classification or diagnostic tools (International Classification of Diseases [ICD]-10 and DSM-IV) to identify individuals eligible for the study (57, 61). The two other studies stated that individuals with first episode psychosis were recruited, but did not provide further details with regards to how this was assessed or diagnosed (37, 40).

The interventions identified in the review were implemented across a range of different settings. Twelve studies examined interventions in an educational setting (school or university) (38, 39, 44, 45, 47–50, 56, 59, 60, 65); other settings included specialist or hospital settings, outpatient or primary care settings and other community settings. Interventions targeting perinatal depression were generally conducted in maternal/childcare centres (four studies) (42, 51, 52, 62). Eight studies reported on the type of geographic setting that programmes were implemented into (43, 44, 52, 55, 61–64). Four studies were conducted in mixed rural and urban settings (43, 52, 62, 63), three were conducted in an urban setting (44, 61, 64) and one study reported on an intervention in a rural setting (55).

The interventions utilised across studies used mixed methodologies. Most commonly (25 of 30 studies), a behaviour therapy intervention was utilised (38, 39, 41–49, 51–56, 58, 60–66). Behaviour therapy interventions were generally based on existing frameworks and therapeutic strategies, such as CBT, problem solving therapy and behaviour activation. In addition, many behaviour therapy interventions incorporated digital health and telehealth in their delivery.

In total, 10 studies reported on interventions that utilised an aspect of digital health (Table 3) (38, 39, 43–45, 51, 52, 56, 59, 63). Eight of these studies utilised online or electronic/computerised versions of behaviour therapy interventions or self-help programmes. In some cases, interventions were designed to be completed by participants in an independent way, however, some programmes made use of messaging services to allow participants to contact lay supporters, facilitators or counsellors (39, 43). Contact between participants and programme facilitators and therapists via phone was also used to encourage adherence (43), provide support and guidance (39, 51), and deliver skills training (51).

**Table 3:** Use of digital health and telemedicine in intervention programmes.

Study name	Geography	Target population	Intervention	Use of digital health/telemedicine
Arjadi 2018 (43)	Indonesia	Individuals aged ≥16 years with MDD	Behavioural activation programme: GAF-ID	Internet-based programme offered through a secure online environment, with adjustments (e.g., replacing videos with illustrations) to improve accessibility for those with poor internet connections. Online environment included a messaging facility to allow communication between participants and lay counsellors. Weekly reminders for participants to log in were provided via online messaging, chat or email. Pre-planned brief phone calls were used to encourage adherence to the programme
Bantjes 2021 (44)	South Africa	University students with anxiety and depression	Group CBT	Group CBT sessions held over Microsoft Teams with an accompanying interactive PDF workbook
Benjet 2023 (45)	Mexico; Colombia	University students with clinically significant anxiety and/or depression	Guided or unguided internet-based CBT	Internet-based CBT with sessions delivered via text, audio, educational video clips, interactive quizzes, exercises and homework. In the guided version, supporters (trained graduate students) provided feedback and personal recommendations to users
Chen 2024 (38)	China	Young people aged 15–25 with comorbid insomnia and MDD	CBT	Electronic CBT for insomnia
Gureje 2019 (52)	Nigeria	Women aged 16–45 years with perinatal depression	Problem-solving treatment	Support, supervision and specialist consultation could be provided by mobile phone. Automated voice messages and calls from primary care providers were used to remind mothers of clinic appointments and homework
Gureje 2022 (51)	Nigeria	Pregnant women aged <20 years with depression	Intervention package including behavioural activation, problem-solving treatment, parenting skills training and parenting skills support	Parenting skills training could be delivered through mobile phone calls and texts
Nejati 2019 (56)	Iran	Young adults aged 18–21 years with moderate to severe depression	AHP	Computerised version of AHP intervention, consisting of 6 tasks (interpreting ambiguous visual stimuli, recognising emotional facial expressions, attention bias modification, completion of ambiguous text, word completion, puzzle task) with positive and negative feedback given to modify interpretation bias
Ofoegbu 2020 (39)	Nigeria	University students with severe depression	ICBT	Initial support session through telephone, regular guidance from therapists through telephone and internet. Video, audio and print materials provided online. Participants worked independently through the programme, guidance therapists provided twice-weekly assistance within the online portal and through email and telephone. Participants could obtain at-need guidance from the therapists through messages within the portal, email or by telephone contact
Osborn 2020 (59)	Kenya	Students aged 13–18 with moderate to severe depression	Digital version of Shamiri self-help programme	Digital self-help version of Shamiri intervention (previously delivered as in person group therapy); content and exercises were adapted to an individual, digital version
Srivastava 2020 (63)	India	Adolescents aged 13–19 years with mild to moderate unipolar depression	“Smart Teen” computer-assisted CBT	Smart Teen web application consisting of 12 CBT sessions. Each session was an interactive multimedia presentation with text, flowcharts, 2D animations, graphics, quizzes and puzzles. Smart Teen platform included practise times, case vignettes, personal diaries and a tool kit of learned skills in addition to the session. Smart Teen was designed to augment standard in-person CBT

AHP, Ambiguous Hallmark Programme; CBT, cognitive behaviour therapy; GAF-ID, Guided Act and Feel-Indonesia; ICBT, internet-based CBT; MDD, major depressive disorder.

Five studies examined behaviour therapy interventions which involved collaborative care between specialists and non-specialists (51–53, 55, 62). In some cases, this involved lay-persons to deliver part of the intervention programme. One study used experienced older women known as “neighbourhood mothers” to provide support and guidance to new adolescent mothers with perinatal depression. These “neighbourhood mothers” were in regular telephone contact with maternal care providers to exchange updates on the progress of the intervention participants (51). Another study trained lay facilitators and community-based health workers to deliver components of a CBT and problem-solving therapy programme designed by psychiatrists; local psychiatrists also participated in in-person supportive sessions for the community-based health workers (53).

In total, 12 studies trained non-specialist healthcare workers (HCWs) or community members to deliver or support the delivery of the intervention. While this was a common practice for interventions targeting MDD, where maternal care providers, students, graduates and community or non-specialist health workers were involved in delivering programmes (42–46, 51–53, 55, 58, 60, 62), all of the interventions targeting schizophrenia used trained specialist HCWs (psychiatrists, psychotherapists and psychiatric nurses) to deliver interventions (37, 40, 57, 61). This likely reflects the settings that interventions were implemented in; for MDD, these included community, educational and primary care settings, while for schizophrenia, interventions were implemented in specialist inpatient or outpatient settings (Table 2).

Two studies which utilised community members or non-specialist HCWs to deliver interventions did not report on the training or supervision for these facilitators (42, 45). Four studies which used non-specialist HCW to deliver mental health literacy interventions reported training and supervision in detail, which included focused training programmes over periods of three days to several weeks, regular supervision and weekly support sessions conducted by specialist healthcare professionals (HCPs) (51–53, 55). In six studies where interventions were provided by lay facilitators (such as students or other peers), training and supervision was more sparsely reported. Four of these studies simply reported that facilitators had been trained in the intervention (44, 46, 58, 60); of the remaining two studies, one reported that facilitators received two days of intensive training (43), while the other reported one week of classroom-based training followed by a two-month clinical internship period (62). Regarding supervision, five studies which used lay facilitators reported that they were supervised by trained professionals, such as licenced clinical psychologists (43, 44, 58, 60, 62).

In 17 studies, a new intervention was compared to standard practise or enhanced usual care (37, 39, 42, 45, 48, 51, 53, 56–58, 60–64). For MDD, standard care usually involved counselling, psychoeducation or referral to existing mental health services; for perinatal depression, usual care generally comprised standard perinatal care, without treatment for depression. Two studies used a waitlist-controlled design (41, 47), in which participants randomised to the control group were waitlisted to initiate the study intervention after a waiting period [3 months after enrolment (41) or after the follow-up evaluation of the treatment group (47)].

Intervention frequency and duration varied between studies. The duration of individual intervention sessions ranged from 30–45 min (43, 52, 62) to 2.5 h (65). The shortest intervention period was a single 1-h session (59); otherwise, interventions were generally provided to participants over a period of one to three months (37–39, 41–45, 47–50, 56, 58, 60, 61, 63, 65, 66) and sessions were most often provided on a weekly basis (39, 42–45, 47, 50–53, 58, 63–66). For intervention programmes which reported that ≥1 session was provided, the number of sessions ranged from three (37, 57) to 16 (52).

Interventions for perinatal depression were generally initiated in the antenatal period and continued for a prespecified period following birth (51–53, 55, 62). In two such programmes, the intensity of the intervention varied based on symptom severity. While total intervention duration was the same for all participants, those with lower EPDS scores (<12) received half the number of intervention sessions as those with higher EPDS scores (≥12), with a longer duration of time between each session (51, 52).

### 3.3 Programme assessment

All 30 studies followed up with participants after the intervention to evaluate effectiveness. The most common follow-up duration was three months post-intervention (11 studies) (37, 41, 42, 45, 47, 49, 50, 52, 61–63) and the longest follow-up time was 14 months (one study of schizophrenia) (40).

All 30 studies assessed intervention outcomes quantitatively from a participant perspective. Across all studies, outcome assessment focused on the mental health condition and symptoms of interest.

Outcomes corresponding to mental health literacy (proximal outcomes) are presented in Table 4 (43, 49, 52–54, 60, 62, 65, 66). Such outcomes included social support, stigma, self-efficacy, cognitive reappraisal and resilience. Assessment of outcomes related to mental health literacy used pre-existing tools and scales [such as the 12-item Discrimination and Stigma scale [DISC-12] (52), Multidimensional Scale of Perceived Social Support [MSPSS] (43, 62) and the Emotional Regulation Questionnaire (66)].

**Table 4:** Programme assessment and outcomes: mental health literacy.

Study name	*N*	Follow-up period for assessment	Outcomes assessed	Assessment methods	Assessment outcomes
MDD					
Arjadi 2018 (43)	Intervention: 120 Comparator: 145	6 months	Fear and avoidance level	Fear questionnaire	Numerical difference in favour of intervention across all assessment timepoints
			Social support	Multidimensional Scale of Perceived Social Support	Numerical difference in favour of intervention across all assessment timepoints
Far 2017 (49)	Intervention: 10 Comparator: 9	3 months	Psychological acceptance/readiness	AAQ-II	Significant increase in acceptance scores between baseline and follow-up in intervention group (*p* = 0.075); no significant increase in acceptance scores between baseline and follow-up in comparator group (*p* = 1)
Gureje 2019 (52)	Intervention: 452 Comparator: 234	12 months postpartum	Stigma	DISC-12	No difference in stigma scores between the 2 groups at 6 months postpartum
Kaaya 2022 (53)	Intervention: 341 Comparator: 395	9 months postpartum	Social support	Duke-UNC Functional Social Support Scale	No difference in social support scores between the intervention and comparator groups at 9 months postpartum
			Self-efficacy	General self-efficacy scale	No difference in self-efficacy scores between the intervention and comparator groups at 9 months postpartum
			HIV-related stigma	Perceived and internalised stigma dimensions	Significant decrease in HIV-related stigma in intervention group compared to comparator group at 9 months postpartum (*p* < 0.05)
Konradt 2018 (54)	61	6 months	Resilience	Resilience scale	Significant increase in resilience scores from baseline (*p* < 0.001)
Savari 2021 (60)	Intervention: 15 Comparator: 15	Post-treatment	Fear of compassion	Fear of Compassion Scale	Significant difference in change from baseline in fear of compassion for others between intervention group and comparator group (*p* = 0.02). No significant differences in change from baseline between intervention group and comparator group across other items (fear of self-compassion, fear of compassion from others)
			Self-criticism and self-reassurance	Forms of Self-Criticising/Attacking and Self-Reassuring Scale	Significant difference in change from baseline between the intervention group and comparator group for hated self (*p* = 0.03) and reassured self (*p* = 0.001). No significant difference change from baseline in inadequate self
			Self-compassion	SCS Short Form	Significant difference in change from baseline between intervention and comparator group for total SCS (*p* = 0.01)
Singla 2021 (62)	Intervention: 423 Comparator: 427	6 months postpartum	Patient activation	PAAS	Significantly higher patient activation in the intervention group compared to the comparator group (*p* < 0.01) at 3 months postpartum
			Social support	MSPSS	Significantly higher social support scores in the intervention group compared to the comparator group (*p* < 0.05) at 3 months postpartum
Xu 2023 (65)	Intervention: 26 Comparator: 22	8 weeks post-treatment	Dispositional mindfulness	FFMQ (Chinese version)	Significantly greater increase in FFMQ scores from baseline in the intervention group compared to the comparator group (*p* < 0.001)
			Compassion	SCS (Chinese version)	Significantly greater increase in SCS scores from baseline in the intervention group compared to the comparator group (*p* < 0.001)
Zemestani 2020 (66)	Intervention: 23 Comparator: 29	16 weeks	Psychological inflexibility and experiential avoidance	AAQ-II	Significant improvements in psychological flexibility in the intervention group compared to the comparator group (*p* < 0.001)
			Cognitive reappraisal	ERQ-R	Adaptive emotion regulation strategies were significantly improved in the intervention group compared to the comparator group (*p* < 0.001)

AAQ, Acceptance and Action Questionnaire; DISC-12, 12-item discrimination and stigma scale; ERQ-R, Emotion Regulation Questionnaire- Reappraisal subscale; FFMQ, Five facet mindfulness questionnaire; HIV, human immunodeficiency virus; MSPSS, Multidimensional Scale of Perceived Social Support; PAAS, PREMIUM Abbreviated Activation Scale; SCS, Self-Compassion Scale; UNC, University of North Carolina.

In studies of interventions for MDD, depression-specific assessments were conducted using standard validated tools, with the most commonly used instruments being BDI-II (10 studies) (39, 47–50, 56, 60, 63, 64, 66) and PHQ-9 (9 studies) (38, 43–45, 53, 55, 58, 62, 65). Some studies used multiple instruments to assess changes in depression following intervention (Table 5). For example, one study used PHQ-9 and a locally adapted version of the Inventory of Depressive Symptomology Self Report to assess depression symptoms, as well as structured clinical interview for DSM-V to assess depression remission (43). One study which recruited adolescents (aged 13 to 19 years) used a child-specific depression scale (Children’s Depression Rating Scale Revised [CDRS-R]) in addition to BDI-II (63). In all but two cases, the same tool (PHQ-9 or BDI-II) used to assess participant eligibility for the study was also used to evaluate changes in depression pre- and post-intervention.

**Table 5:** Programme assessment and outcomes.

Study name	N	Follow-up period for assessment	Outcomes assessed	Assessment methods	Assessment outcomes
MDD					
Alavi 2013 (41)	Intervention: 15 Comparator: 15	3 months	Suicidal ideation	SSI	Significant decrease from baseline in SSI, BHI and BDI scores (all *p* < 0.001) at 3 months in intervention group; significant difference in SSI, BHI and BDI score (all *p* < 0.001) between intervention and comparator group at 3 months
			Probability of hopelessness	BHI	
			Depression symptoms	BDI	
Alhusen 2021 (42)	Intervention: 30 Comparator: 30	12 weeks postpartum	Depression symptoms	EPDS	Numerically greater decrease in EPDS score between baseline and 12 weeks postpartum in intervention group compared to comparator group
			Mother–child attachment	MFAS	Larger change from baseline in MFAS score between baseline and 36 weeks gestation in intervention group compared to comparator group
			Maternal sensitivity	NCAST-Feeding scale	Scores suggested more favourable levels of mother–child interaction in intervention group compared to comparator group
Arjadi 2018 (43)	Intervention: 120 Comparator: 145	6 months	Depression symptoms	PHQ-9	Self-reported depression symptoms were lower in the intervention group than the comparator group at 6 months of follow up (*p* = 0.007)
				Inventory of Depressive Symptomology Self-Report (Indonesian Version)	Statistically significantly lower scores in the intervention group compared to the comparator group at 6 months of follow-up (*p* = 0.007)
			Depression remission	SCID-5	Participants in the intervention group had a 50% higher chance of depression remission at 10 weeks than participants in the comparator group
			QoL	WHOQoL-Bref	Higher in intervention than in comparator across all assessment timepoints
Bantjes 2021 (44)	158	Post-treatment	Aggregate symptom score	PHQ-9; GAD-7	Statistically significant decrease in scores after intervention compared with baseline (*p* < 0.001)
			Response and remission	Clinically significant decreases in symptoms, remission rates (PHQ-9, GAD-7) and clinical deterioration	Statistically significant decrease in scores after intervention compared with baseline (*p* < 0.001)
			Treatment satisfaction	Feedback from participants	Most participants were satisfied overall with the intervention (113/125, 90.4%)
Benjet 2023 (45)	Guided ICBT: 445 Self-guided ICBT: 439 Comparator: 435	3 months	Depression remission	PHQ-9	Statistically significantly higher remission rate in the guided ICBT group than the self-guided ICBT group (*p* = 0.001) and comparator group (*p* = 0.004)
				ITR using machine learning methods	Significantly higher probability of remission in the guided ICBT group than the self-guided ICBT group (*p* = 0.001) and comparator group (*p* < 0.001)
			Depression and anxiety symptoms	PHQ-ADS	Numerically lower symptom scores in the guided ICBT group compared with the self-guided ICBT group and comparator group
Chen 2024 (38)	Intervention: 56 Comparator: 57	6 months	Depression symptoms	PHQ-9	Significant decrease in depressive symptoms, evaluated by PHQ-9 scores, from baseline to 6-month follow-up (*p* < 0.001); numerical decrease in depressive symptoms in intervention group compared to comparator group
			Insomnia	ISI	Less severe insomnia symptoms and reduced risk of insomnia at 6-month follow-up in intervention group compared to comparator group
Church 2012 (46)	Intervention: 9 Comparator: 9	Post-treatment	Depression symptoms	BDI	Substantially lower post-treatment BDI scores in the intervention group compared to the comparator group. Following intervention, mean participant scores were in the non-depressed range on BDI
Eseadi 2022 (47)	Intervention: 34 Comparator: 33	3 months	Depression symptoms	BDI-II	Substantial decrease in BDI-II scores at 3 months and significant difference in BDI-II scores between the intervention group and the comparator group (*p* < 0.05)
Ezeudu 2020 (48)	Intervention: 12 Comparator: 11	Post-treatment	Depression symptoms	BDI-II	Significant decrease in depression scores from baseline to follow-up (*p* < 0.001) and significant difference in depression scores between the intervention group and comparator group (*p* < 0.001)
Far 2017 (49)	Intervention: 10 Comparator: 9	3 months	Depression symptoms	BDI-II	Significant decrease in depression scores between baseline and follow-up in both the intervention (*p* = 0.04) and comparator groups (*p* = 0.007). No significant difference in depression score at follow-up in intervention group compared to comparator group
			Dysfunctional attitudes	DAS	Numerical decrease in dysfunctional attitude score between baseline and follow-up in intervention group; significant decrease in dysfunctional attitude score between baseline and follow-up in comparator group (*p* = 0.05)
Fereydouni 2022 (50)	Intervention: 30 Comparator: 30	12 weeks	Depression symptoms	BDI-II	Significantly greater decrease in BDI-II scores in intervention group compared with comparator group (*p* < 0.001)
			Depression, anxiety and stress symptoms	DASS-42	Significantly lower scores in intervention group compared with comparator group for depression, anxiety, stress and total symptom score (all *p* < 0.001)
Gureje 2019 (52)	Intervention: 452 Comparator: 234	12 months postpartum	Depression symptoms	EPDS	Numerically higher proportion of participants recovered from depression in the intervention group compared with the comparator group at 6 months. Significantly lower mean EPDS score at 12 months postpartum in the intervention group compared with the comparator group (*p* = 0.012)
			Functional impairment/disability	WHODAS	Statistically significantly lower WHODAS score at 6 months postpartum in the intervention group compared to the comparator group (*p* = 0.045); numerically lower WHODAS score at 12 months postpartum in the intervention group compared to the comparator group
			Pharmacological treatment rate	Proportion of mothers prescribed with pharmacological treatment for depression	No participants in either group were prescribed antidepressants
Gureje 2022 (51)	Intervention: 141 Comparator: 101	6 months postpartum	Depression symptoms	EPDS	A significantly higher proportion of participants achieved remission from depression at 6 months postpartum in the intervention group compared with the comparator group (*p* = 0.005). Significantly lower depression scores in the intervention group compared to the comparator group at 6 months postpartum (*p* = 0.003)
			QoL	WHOQoL-Bref	No significant difference across physical, psychological, social or environmental domain of WHOQoL-Bref between intervention and comparator groups at 6 months postpartum
			Functional impairment/disability	WHODAS	No significant difference between intervention group and comparator group at 6 months postpartum
Kaaya 2022 (53)	Intervention: 341 Comparator: 395	9 months postpartum	Depression symptoms	PHQ-9	Significantly lower risk of MDD (*p* < 0.001) and significantly lower PHQ-9 scores (*p* = 0.012) in the intervention group compared to the comparator group at 9 months postpartum
			HIV-related stigma	Perceived and internalised stigma dimensions	Significant decrease in HIV-related stigma in intervention group compared to comparator group at 9 months postpartum (*p* < 0.05)
Konradt 2018 (54)	61	6 months	Depression symptoms	HDRS	Significant decrease in depression scores from baseline (*p* < 0.001)
			Anxiety symptoms	HARS	Significant decrease in anxiety scores from baseline (*p* < 0.001)
Nakku 2021 (55)	153	6 months	Depression symptoms	PHQ-9	Significant decrease in PHQ-9 score from baseline (*p* < 0.0001) with 93.7% of participants experiencing a clinical response (≥50% reduction in symptom score)
			Functional impairment/disability	WHODAS	Significant reduction in WHODAS score from baseline (*p* < 0.0001)
Nejati 2019 (56)	Intervention: 11 Comparator: 11	Post-intervention	Depression symptoms	DASS depression scale	Significant reduction in depression symptoms in the intervention group compared to comparator group (*p* < 0.01)
				BDI-II	
Ofoegbu 2020 (39)	Intervention: 56 Comparator: 58	4 weeks	Depression symptoms	BDI-II	Substantial decrease in BDI-II scores in the intervention group and significant reduction in depression between the intervention and comparator groups at 4 weeks (*p* = 0.000)
Olashore 2023 (58)	Intervention: 25 Comparator: 25	24 weeks	Depression symptoms	PHQ-9	Significant decrease in depression scores in the intervention group across follow-up period (*p* < 0.05). Significant difference in depression symptoms at 5 weeks post-intervention in the intervention group compared to the comparator group (*p* = 0.001)
			HIV treatment adherence	VAS	Significant increase in treatment adherence across the follow-up period (*p* > 0.05) in the intervention group. Significantly higher adherence scores in the intervention group compared with the comparator group at 5 weeks post-intervention (*p* = 0.001)
			Therapy satisfaction	CSQ	Level of satisfaction with the intervention was high at 5 weeks post-intervention
Osborn 2020 (59)	Intervention: 28 Comparator: 28	2 weeks	Depression symptoms	PHQ-8	Decrease in depression symptoms from baseline in the intervention group and greater decreases in depression symptoms from baseline in the intervention group compared with the comparator group
			Intervention acceptability	Questionnaire	No significant differences in acceptability between the intervention and comparator group
Savari 2021 (60)	Intervention: 15 Comparator: 15	Post-treatment	Depression symptoms	BDI-II	Substantial reduction in BDI-II score in intervention group post-treatment. Change in BDI-II scores from pre- to post-treatment were significantly different between intervention group and comparator group (*p* = 0.006)
			Anger	ARS	Change from pre- to post-treatment between the intervention and comparator groups were significantly different for angry afterthoughts (*p* < 0.05), thoughts of revenge (*p* = 0.05), angry memories (*p* < 0.01) and understanding of causes (*p* < 0.01)
Singla 2021 (62)	Intervention: 423 Comparator: 427	6 months postpartum	Depression symptoms	PHQ-9	Significant difference in PHQ-9 scores between intervention group and comparator group at 3 months (*p* < 0.001) and 6 months (*p* < 0.10) postpartum
			Mother–child attachment	Maternal Postnatal Attachment Scale	No significant differences in mother–child attachment between intervention and comparator at 3 months postpartum
Srivastava 2020 (63)	Intervention: 11 Comparator: 10	12 weeks	Depression symptoms	BDI-II	Average depression scores were significantly lower in the intervention group compared with the comparator group (BDI-II *p* = 0.04; CDRS-R *p* = 0.03; CGI-S *p* = 0.02; CGAS *p* = 0.002). A greater number of participants in the intervention group achieved ≥50% reduction in depressive symptoms and general functioning than in the comparator group
				CDRS-R	
				CGI-S	
				CGAS	
			Acceptability	35-litem Likert scale survey	Survey responses demonstrated a high level of acceptability for the Smart Teen intervention
			Time spent by therapist	N/A	Time spent by therapist was longer for comparator group than for intervention group
Toth 2013 (64)	Intervention: 99 Comparator: 29	8 months post-treatment	Depression symptoms	BDI-II	Significantly greater decreases in depression over time in the intervention group compared to the comparator group (BDI-II *p* = 0.005; HRSD-R *p* = 0.038)
				HRSD-R	
Xu 2023 (65)	Intervention: 26 Comparator: 22	8 weeks post-treatment	Depression symptoms	PHQ-9	Significantly greater reduction in PHQ-9 scores from baseline in the in the intervention group compared to the comparator group (*p* < 0.001)
			Anxiety symptoms	GAD-7	Significantly greater reduction in GAD-7 scores from baseline in the in the intervention group compared to the comparator group (*p* < 0.001)
			Sleep quality	PSQI	Significantly greater reduction in PSQI scores from baseline in the in the intervention group compared to the comparator group (*p* < 0.001)
Zemestani 2020 (66)	Intervention: 23 Comparator: 29	16 weeks	Depression symptoms	BDI-II	Significant improvements in depression symptoms in intervention group compared to the comparator group (*p* < 0.001)
			Psychological wellbeing	SPWB	Psychological wellbeing was significantly improved in the intervention group compared to the comparator group (*p* < 0.001)
Schizophrenia					
Correa-Oliveira 2022 (40)	237	14 months	FEP outcomes	Clinical assessment	NR
Mlay 2022 (37)	NR	Month 1 and Month 3	Intervention acceptability	Questionnaire	NR
			Schizophrenia relapse	Re-admission to hospital	NR
			Medication adherence	VAS	NR
			QoL	Lehman Quality of life instrument	NR
			Personal and social function	PSP scale	NR
Ngoc 2016 (57)	Intervention: 30 Comparator: 29	6 months	QoL	Quality of Life Enjoyment and Satisfaction Questionnaire	Intervention resulted in non-statistically significant improvements in patient-reported QoL and stigma
			Schizophrenia-related stigma	Stigma Towards Schizophrenia scale	
			Therapy satisfaction	4-point Likert scale tool developed during study	Patient-reported satisfaction was numerically higher in the intervention group compared to comparator group
She 2016 (61)	Intervention: 30 Comparator: 30	1 year	Consistency between self and experience	SCCS Chinese Version	Statistically significant differences in favour of intervention up to 3 months post intervention, however, no significant difference was observed at 1 year of follow-up
			Schizophrenia symptoms	PANSS Chinese Version	

ARS, Anger Rumination Scale; BDI, Beck Depression Inventory; BHI, Beck Hopelessness Inventory; CDRS-R, Children’s Depression Rating Scale Revised; CGAS, Children’s Global Assessment Scale; CGI-S, Clinician’s Global Impression Scale-Symptom Severity Subscale; CSQ, Client Satisfaction Questionnaire; DAS, Dysfunctional Attitude Scale; DASS, Depression, Anxiety and Stress Scale; EPDS, Edinburgh Postnatal Depression Scale; GAD-7, Generalised Anxiety Disorder-7; HARS, Hamilton Anxiety Rating Scale; HDRS, Hamilton Depression Rating Scale; HRSD-R, Hamilton Rating Scale for Depression-Revised; ICBT, internet-based cognitive behaviour therapy; ISI, Insomnia Severity Index; ITR, individualised treatment rule; MFAS, Maternal-Foetal Attachment Scale; N/A, not applicable; NCAST, Nursing Child Assessment Satellite Training; NR, not reported; PANSS, Positive and Negative Syndrome Scale; PHQ, Patient Health Questionnaire; PHQ-ADS, Patient Health Questionnaire-Anxiety and Depression Scale; PSP, Personal Social Performance; PSQI, Pittsburgh Sleep Quality Index; QoL, quality of life; SCCS, Self-Consistency and Congruence Scale; SCID-5, Structured Clinical Interview for DSM-V; SPWB, Scales of Psychological Well-Being; SSI, Scale for Suicidal Ideation; WHODAS, World Health Organisation Disability Assessment Scale; VAS, visual analogue scale; WHOQoL-Bref, World Health Organisation Quality of Life Brief Version.

The four studies of interventions for individuals with schizophrenia all used different instruments and methods to assess programme effectiveness (Table 5). Similar to the assessment of depression, pre-existing validated tools, such as the Positive Negative Syndrome Scale (PANSS) and the Self-Consistency and Congruence Scale (SCCS) (61), were used to assess symptoms, with appropriate translations being utilised as required. Two studies also used clinical assessments (follow-up diagnosis or relapse) to evaluate the success of an intervention (37, 40).

Across studies of interventions for both MDD and schizophrenia, other measures for mental health-related outcomes were also used. Assessments again utilised appropriate validated tools and instruments; the specific tools used to assess outcomes varied between studies according to their overall objectives. For example, studies examining interventions for perinatal depression used tools to assess mother–child attachment while studies examining interventions aiming to alleviate suicidality used tools to evaluate suicidal ideation and self-harm. Other studies additionally evaluated participant mental health more holistically by utilising instruments to evaluate anxiety and stress, including Generalised Anxiety Disorder-7 (GAD-7), Depression, Anxiety and Stress Scale-42 (DASS-42) and the PHQ Anxiety and Depression Scale (PHQ-ADS, which combines PHQ-9 and GAD-7) (67). Finally, some studies assessed overall participant quality of life (QoL) using general QoL scales such as WHOQoL Brief Version (WHOQoL-Bref).

Only two studies assessed interventions from a healthcare system perspective (52, 63). In one study of a problem-solving therapy intervention for women with perinatal depression in Nigeria, the proportion of participants who were prescribed pharmacological treatment was reported (52). In one study of computer-assisted CBT in India, the therapist time required to deliver computer-assisted CBT was evaluated and compared with the therapist time required to deliver treatment as usual (63). No studies evaluated outcomes from a societal perspective.

The majority of studies included in the review examined short-term outcomes, with a focus on the improvements in mental health symptoms over a short timeframe. Twenty-one studies reported follow-up durations of 6 months or less and 11 studies reported follow-up durations of 3 months (12 weeks) or less (37–39, 41–43, 45–47, 49–51, 54, 55, 57–59, 62, 63, 65, 66). Only three studies reported follow-up of at least one year (40, 52, 61). Four studies did not clearly report the duration of follow-up (44, 48, 56, 60).

### 3.4 Programme outcomes

Among measures of mental health literacy (proximal outcomes), statistically significant improvements associated with various interventions were observed in resilience (at 6 months of follow-up) (54), self-criticism and reassurance, self-compassion (measured post-treatment) (60), dispositional mindfulness and compassion (at 8 weeks post-treatment) (65), emotional regulation strategies (at 16 weeks of follow-up) (66) and psychological acceptance/readiness at 3 months follow-up (49), compared to baseline and/or control groups. Studies reporting on social support showed more variable results, with one study (62) reporting significant improvements in social support via the MSPSS in women receiving behaviour activation therapy through the Think Healthy Programme at three months postpartum, compared to controls who received enhanced usual care. Another study (43) reported numerical improvement in social support via the MSPSS in those having received behaviour activation therapy compared to the control group at six month follow-up, whereas a third study (53) showed no significant differences in social support via the Duke-UNC Functional Social Support Scale between those having received problem solving therapy (PST) combined with CBT and those with enhanced usual care at nine months postpartum. Interestingly, in the one study assessing mental health stigma (52), no difference in this outcome via the DISC-12 discrimination and stigma scale was found between the intervention group receiving PST and the control group receiving enhanced usual care at six months postpartum.

Most studies primarily focused on symptomatic improvement (distal outcomes). In most comparative studies, statistically significant improvements in depression symptoms were reported in the intervention group compared to the control group (39, 41, 43, 45–48, 50–53, 56, 58–60, 62–66), demonstrating that the majority of interventions for MDD identified in this review could be considered effective in terms of distal mental health outcomes (Table 5). Non-significant results were found in some studies which compared different versions of the same intervention or compared different interventions in previously untreated populations (38, 45, 49). Across other measures for mental health-related outcomes in individuals with MDD and schizophrenia, statistically or numerically positive improvements were also observed.

Across the 30 included studies, only 13 clearly reported outcomes measured ≥1 month after the end of the intervention (38, 42, 43, 45, 49, 51, 52, 55, 58, 61, 64–66). Among these studies, duration of follow-up after the end of the intervention ranged from 1 month (58) to 12 months (61) but only two studies measured outcomes at ≥6 months post-intervention (61, 64). Statistically significant improvements in mental health symptoms were observed across post-intervention follow-up of up to eight months (38, 42, 43, 45, 49, 51, 52, 55, 58, 64–66). While the duration of follow-up was limited in the majority of cases, 10 of the 13 studies that reported post-intervention follow-up of ≥1 month observed statistically significant benefits associated with the intervention up to the end of the follow-up period (38, 45, 49, 51, 52, 55, 58, 64–66). One study in patients with schizophrenia noted a statistically significant difference in favour of the intervention at 3 months of follow-up, but no significant difference at 12 months (61). Based on the included studies, no clear trends were observed between the frequency of interventions, overall intervention duration and intervention effectiveness in improving mental health outcomes, however, conclusions were also limited by short follow-up durations.

### 3.5 Replicability and scalability

Eight studies subjectively discussed the replicability of an intervention (40, 43, 50, 52, 57, 59, 60, 62). The authors of four studies, which examined interventions including problem solving therapy for women with perinatal depression, behaviour activation for people with depression, early intervention for people with first episode psychosis and a family education programme for young people with schizophrenia suggested that the success of their programmes could be replicated in similar resource-limited settings (40, 43, 52, 57). In addition, one study noted the potential value of assessing of the cost-effectiveness of the intervention during any future replication studies (43).

The challenges of replicating programmes were also discussed in two studies. One study highlighted potential replicability issues in the context of digitally delivered interventions in resource-limited settings. The availability of appropriate technology in educational settings in Kenya was raised, highlighting a wider consideration in ensuring that the intended audience would be able to access a digitally delivered intervention (59). One study highlighted the importance of the local context to the success of an intervention, noting that their trial was conducted in an area with “relatively high literacy levels”. Authors noted that the acceptability of their intervention may be different among populations in poorer socio-demographic circumstances and in different health system contexts’, highlighting the importance of considering local adaptation when replicating mental health interventions (62).

Eight studies subjectively discussed the scalability of the intervention under investigation. Six studies stated that the characteristics of the interventions investigated made them potentially suitable for large-scale dissemination. Reasons for this conclusion included the intervention being conducted online, incorporation of digital health allowing more individuals to be reached with the same resource as more traditional care, and the authors considering that the intervention was cost-efficient and cost-effective (37, 43, 44, 51, 59, 62). In two studies, the authors stated that the intervention had potential for scalability but did not elaborate on specific aspects of the intervention facilitating this (39, 55).

## 4 Discussion

This SLR identified 30 studies reporting on public health interventions for mental health and mental health literacy in young people with MDD or schizophrenia in resource-limited settings. Studies were included from countries in Asia, Africa, South America and North America, offering insights across different global contexts. The majority of the studies included in the review were of good quality, indicating a generally high level of confidence in the conclusions drawn from this SLR.

Almost all studies explored the implementation of a behaviour therapy, education or self-care programme. Many interventions were a mix of multiple types, such as combining behaviour therapy and digital health. In particular, the number of studies involving the use of telehealth as part of an intervention (e.g., electronic or online programmes) indicates this as a key area for exploration when designing interventions for LMICs.

The interventions identified as part of this SLR all have the potential to improve mental health literacy as part of improving mental health outcomes. In particular, several interventions and outcomes could be conceptually mapped to components of Jorm’s mental health literacy framework. Interventions incorporating behavioural activation, structured skills practise and psychoeducation [e.g., internet-based behavioural activation (43), the Think Healthy behavioural activation programme (62) and PST/CBT (52, 53), among others] targeted self-management strategies that can be conceptually mapped to Jorm’s domain of “knowledge and beliefs about self-help interventions”. Although none of the studies assessed this exact domain, improvements in patient activation (62), psychological flexibility and cognitive reappraisal (49, 66), mindfulness and self-compassion (65), self-criticism/self-reassurance and fear of compassion (60) can indicate greater engagement with, and perceived usefulness of, self-directed coping strategies. Similarly, changes in self-relating processes (60) map indirectly onto Jorm’s “attitudes which facilitate recognition and appropriate help-seeking”, by potentially lowering internal and interpersonal barriers to seeking care. In addition studies which explicitly evaluated stigma and self-efficacy (52, 53) clearly link to expanded mental health literacy frameworks which account for the relationship between stigma and mental health knowledge (21).

Other interventions and outcomes identified in this SLR may align more intuitively with broader behaviour change frameworks, that can sit alongside Jorm’s mental health literacy framework. In particular, these interventions align with newer concepts incorporated into mental health literacy, such as self-efficacy and the more encompassing idea of “understanding how to obtain and maintain positive mental health” (21). Behaviour change frameworks such as COM-B (68) emphasise capability (e.g., cognitive and emotional skills), opportunity (e.g., social support) and motivation (e.g., reduced stigma, greater self-compassion) which may be central to how people put mental health knowledge into practise. From this perspective, these outcomes can act as drivers of engagement with both self-help and professional care for mental health conditions (69).

Despite the potential of these interventions for improving mental health literacy, this review identified a lack of consistent and explicit measurement of mental health literacy as an outcome in the design of public health intervention trials, indicating an important consideration in the design of future trials.

Another key issue in mental health care in resource-limited settings is healthcare workforce capacity (16, 17) and the mental health treatment gap in LMICs is usually attributed to a lack of specialist HCWs (30). It has been suggested that substantially increasing the pool of specialist mental health providers is not a feasible or appropriate model to follow for resource-limited healthcare systems (30). Instead, other human resources (such as lay individuals with lived experience, peers, community health volunteers, traditional healers and educators) should be leveraged to address the gap (30). Collaborative care was explored as an intervention strategy across several studies and mainly involved task-shifting of mental health care activities to non-specialist HCWs, a model that can help to alleviate the burden on existing HCWs. The effectiveness of programmes identified in this review which trained lay persons or HCWs without a background in mental health care to deliver or help facilitate an intervention demonstrates the potential of this idea in addressing the mental health gap in resource-limited settings.

A substantial majority of studies identified in the SLR only investigated outcomes from the participant perspective. While this provides insight into tools which can be used in these contexts to evaluate the success of programmes in terms of participant mental health and mental health literacy outcomes, there is a considerable gap in understanding the wider healthcare system and societal impact of these programmes. Understanding the impact of mental health and mental health literacy interventions for young people on the healthcare system and wider society, such as impacts on overall disease burden and healthcare capacity, would enable a better understanding of the wider value to different stakeholders and the public health benefit of different interventions.

### 4.1 Key considerations for programme design

**Figure 2:**
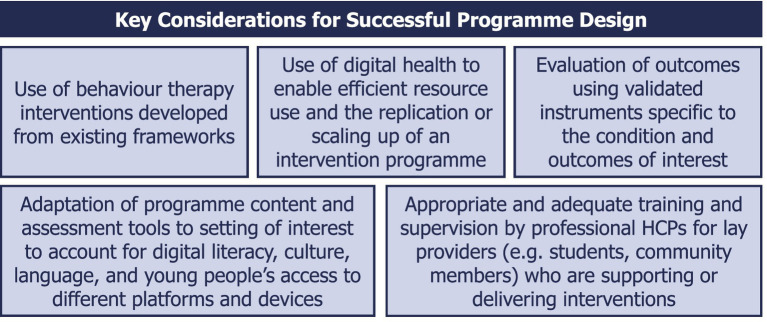
Key considerations for programme design.

An aim of this SLR was to identify key considerations (Figure 2) for designing intervention programmes to improve the mental health and mental health literacy of young people with MDD or schizophrenia. A lack of assessment of mental health literacy outcomes across the majority of studies identified in this review suggest that public health interventions that aim to not only improve mental health outcomes but literacy as well, should incorporate explicit measurements of mental health literacy alongside clinical measures, e.g., structured assessment of knowledge about self- and professional help, help-seeking intentions and knowledge of how to seek information.

In terms of the interventions themselves, the studies captured in this review successfully utilised behaviour therapy interventions based on existing frameworks to achieve positive mental health outcomes, assessed from the perspective of participants. Such interventions were implemented across regions of interest, suggesting they are universally effective and highly replicable, provided they are adequately adapted to the local context (43, 45, 52).

Digital health was widely highlighted as a feature which enabled programmes to be replicated and scaled in resource-limited settings. Digital formats can reduce the cost and resource use associated with new interventions (70), however, the digital literacy of the population and the platform through which the intervention is delivered need to be carefully considered for the setting of interest (62).

In relation to the benefits of digital health for resource-limited settings, systematic reviews of digital health interventions noted that the large majority of digital health interventions are cost-effective, with most digital health interventions being associated with higher quality-adjusted life years (QALYs) while being cost-saving compared to the alternative. In addition, the benefits of utilising digital health interventions to reach rural populations are often underlined as a key consideration for resource-limited settings (71).

One study identified in this review demonstrated that addition of computer-assisted CBT to usual primary care resulted in significant reduction in depression symptoms and general functioning (63). Improving mental health outcomes through the addition of digital health interventions has been demonstrated in other contexts. The “Smiling is fun” internet-delivered CBT-based self-help programme for depression has been investigated as an addition to treatment-as-usual in European primary care settings, and results in significantly improved mental health outcomes over 15 months (72, 73). Furthermore, an economic evaluation of the same programme concluded it to be a cost-effective approach to improving clinical outcomes (73).

In addition, collaborative care models, task shifting and training of peers, students and graduates, or HCWs without a background in mental health care were widely used to support and deliver effective interventions targeting MDD (42–46, 51–53, 55, 58, 60, 62). Use of these alternative human resources was noted as being a low-cost solution which can enable the scaling up of interventions (62). Similarly, implementing an internet-based intervention supported by lay counsellors was highlighted as an approach that could help to bridge the mental health gap in LMICs (43). Indeed, using lay counsellors to provide effective mental health interventions has been widely accepted in these settings (74, 75).

However, it is still vital to ensure that lay providers are supported by sufficient specialist HCP capacity, and that lay providers are appropriately trained and supported in the delivery of interventions. Prior to programme initiation, lay providers should receive adequate and appropriate training from professional providers. In addition, supervision and support from specialist HCPs is required for the duration of an intervention programme (76).

These recommendations for training and supervision of lay providers are echoed by a qualitative systematic review of lay counsellor services in South Africa. This review noted that in a number of cases, lay councillors showed a lack of fidelity to the counselling model that they had been trained to deliver, likely in part due to lack of supervision. In addition, it was noted that training was usually provided as a one-off, and refresher training or training updates were rarely provided. Finally, a number of studies recommended ensuring psychological support structures are in place for lay providers, to improve quality of provision and ensure lay HCW do not experience burn-out (77).

The studies identified in this review generally reported short follow-up times, most commonly evaluating outcomes at the end of the intervention period. Less than half of the included studies clearly reported outcomes measured at least 1 month after the end of the intervention (38, 42, 43, 45, 49, 51, 52, 55, 58, 61, 64–66). While short-term evaluation of outcomes is valuable for assessing the immediate effectiveness of interventions, this results in a gap in the understanding of the long-term impact and sustainability of mental health literacy programmes. While 10 studies reporting post-intervention follow-up of ≥1 month demonstrated that the mental health benefits associated with the intervention were maintained across the follow-up period (38, 45, 49, 51, 52, 55, 58, 64–66), the duration of follow-up remained limited in the majority of cases. It is therefore unclear how long these significant benefits may be sustained for. Future programmes should plan for both short- and long-term follow-up, ideally of ≥12 months post-intervention, in order to address these gaps.

When evaluating participant outcomes, validated scales should be used to assess mental health before and after intervention. PHQ-9 and BDI-II are suitable and widely used scales for evaluating MDD. Assessing other outcomes of interest, such as resilience and self-efficacy, can add value to programme assessment; evaluation tools should be carefully chosen to align with the aims of the intervention. In the studies included in this review, pre-existing validated scales were successfully used across different geographies and contexts. Studies often utilised existing adaptations of these tools [such as the Indonesian version of the Inventory for Depressive Symptomology Self Report (43), Chinese version of the SCCS (61), Luganda version of the PHQ-9 (55)] appropriate for the local language and cultural context. One study also reported using an adapted version of the PHQ-9 which the authors considered more appropriate for use in their Kenyan school setting (59). The appropriateness of individual tools for use in a particular setting and participant population, including ensuring the concepts assessed are contextually relevant and are easily understood by participants, is an essential consideration. Depending on the objective of the intervention, an existing pre-validated tool may not be available to measure specific concepts. Nevertheless, evidence from the studies identified in this review demonstrates that existing assessment tools can be used across a wide range of contexts, which may reduce the resource required for programme evaluation.

### 4.2 Strengths and limitations

The SLR was performed in line with best practice as recommended by the Cochrane Handbook for Systematic Reviews of Interventions (78). The protocol was prospectively developed and registered with PROSPERO before the initiation of the review. A comprehensive breadth of sources, including electronic databases, non-English databases, grey literature websites, reference lists of relevant SLRs, trial registries and conference proceedings, to identify relevant literature. There were no language restrictions, ensuring all relevant data from different regions could be captured. In order to prioritise the most recent evidence published since the WHO Youth Mental Health declaration (10), only studies published in or since 2010 were eligible for inclusion.

Studies identified as part of this review were generally found to be of high quality when assessed using the AHFMR checklist. Of note, the results of the two studies included in this SLR which scored ≤0.75 on quality assessment and reported programme assessment outcomes did not substantially differ from the results of higher quality studies included in this SLR, reporting a significant decrease in mental health symptoms at follow-up compared to baseline (38, 39).

Nevertheless, this SLR is associated with some limitations. Though studies were generally of good quality, some shortcomings were identified in the quality assessment. Many studies did not report how confounding was accounted for and, in studies where this would have been possible, randomisation and blinding were not always performed or fully reported. These potential concerns need to be taken into account when interpreting the results of the studies identified here.

This SLR also identified some gaps within the evidence base. Of particular note, few studies evaluated the impact of programmes on mental health literacy specifically, instead focusing on measuring changes in mental health symptoms. While the interventions captured in the SLR were interventions which can improve mental health literacy, mental health literacy was not explicitly measured in the majority of cases. Instead, programmes focused more explicitly on evaluating mental health symptoms, therefore understanding programme outcomes in terms of mental health improvements. It is therefore challenging to understand the extent to which the identified interventions improved the mental health literacy of programme participants.

Additionally, interventions were rarely examined from a healthcare system or wider societal perspective, precluding an examination of the broader impact of implementing new mental health literacy interventions. In addition, as the majority of publications focused on mental health outcomes, few studies reported the full details of programme implementation.

Furthermore, not all of the studies identified focused on interventions for young people aged 15–29 years. In some cases, eligibility criteria allowed older or younger participants to be recruited. As a result, some of the identified studies are likely to present more general interventions for addressing mental health and mental health literacy in people with severe mental health conditions, rather than focusing specifically on interventions designed to be effective for young people.

The findings of this SLR should be interpreted in the context of the heterogeneity of settings identified. This SLR included studies conducted in 17 different countries, including 16 LMICs and resource-limited settings in one HIC. One study also noted within-country heterogeneity as important (62), and this may be particularly important where substantial urban–rural divide still exists. While overarching lessons for programme design can be drawn from the literature, the importance of adaptation to the local context remains key.

Finally, an assessment of publication bias was not carried out as part of this SLR. While many of the included studies reported statistically significant positive results in favour of the intervention compared to the control, unfavourable results or unsuccessful programmes may not be published or disseminated. As a result, aspects of programme design which result in an intervention being less successful may not be identified.

### 4.3 Future research

This review identified key evidence gaps in understanding the benefits of mental health literacy programmes for improving the mental health of young people in LMICs. In particular, when understanding how best to design intervention programmes for resource-limited settings, intervention sustainability, longevity and broader economic impact are also important to consider.

The healthcare system and societal impact of programmes were not often evaluated in the studies identified in this review. Given the resource constraints of the settings of interest, it is vital to understand the return on investment that mental health literacy interventions can provide. Incorporating economic evaluation, from both healthcare system and societal perspectives, into future mental health literacy programmes would allow this important topic to be investigated in further detail. This also reflects the growing calls globally to address the economic issues around mental health. For example, the recently formed Coalition for Mental Health Investment frames the need to address global mental health as an economic prospect and promotes the importance of investment in mental health from the perspective of reducing healthcare costs and increasing economic productivity, on top of improving wellbeing (79).

Additionally, it is key to add to the limited evidence base around the cost-effectiveness and economic impact of mental health programmes in LMICs; a recent systematic review of economic evaluations for mental health prevention and promotion interventions highlighted the limited evidence currently available for LMICs (80). In particular, further investigation of the cost-effectiveness of utilising digital health to enhance programme outcomes would be of value.

A major evidence gap identified in this review is the lack of studies reporting on mental health literacy outcomes. This evidence gap has also been observed in other recent reviews of mental health literacy in young adults (81). While this review identified many public health interventions with the potential to improve the mental health of young people, the lack of mental health literacy evaluation means it is difficult to conclude if mental health improvements were facilitated by improvements in mental health literacy. There is a clear need for further research to investigate mental health literacy outcomes for programmes targeting young people aged 15–29 years old in resource-limited settings. As with the evaluation of mental health outcomes, assessment of mental health literacy outcomes in future programmes should utilise pre-existing validated tools, adapted to the local setting as needed.

Finally, as discussed above, this review identified a key gap in understanding the long-term impacts of mental health literacy interventions for young people, with the majority of studies reporting a short period of follow-up. Future work should ensure that the long-term impact of such interventions is investigated, in order to understand the duration of an intervention’s effect. This can also be combined with investigating the relationship between intervention duration and the longevity of the intervention’s impact, in order to optimise outcomes.

In the context of resource-limited settings, intervention sustainability is a key aspect of ensuring that interventions can be delivered over a long period of time, maximising their benefit to individuals, the community and the healthcare system. Related to understanding the cost-effectiveness of mental health literacy interventions, ensuring healthcare system sustainability should be a key goal of future intervention programmes and there is a clear need for further research on the sustainability of mental health literacy interventions for individuals with MDD or schizophrenia in resource-limited settings.

## 5 Conclusion

This SLR identified 30 studies investigating public health interventions aiming to improve the mental health and mental health literacy of young people in resource limited settings. The findings suggest that behavioural therapy, education and self-care programmes, enabled by digital tools, have the potential to improve mental health outcomes in such settings. However, limited evidence was identified regarding the mental health literacy outcomes of such programmes. The use of community HCW can alleviate concerns with healthcare workforce capacity, but adequate training and supervision by specialist HCPs is still necessary. In addition, this study identified key aspects of programme design that enabled appropriate evaluation of the mental health outcomes of interventions from the participant perspective, such as the use of appropriate validated tools, as well as drivers and barriers to the replicability and scalability of interventions (e.g., use of digitally delivered interventions available on appropriate platforms and appropriate adaptation to local context).

However, evidence gaps remain, particularly around impact on mental health literacy, and sustained improvements over the long term and the impact of programmes on the healthcare system and wider society. Further research is needed to more fully understand the potential impact of such programmes on aspects such as healthcare capacity and sustainability, and the workload of HCWs.

## Data Availability

The original contributions presented in the study are included in the article/Supplementary material. Further inquiries can be directed to the corresponding author.

## Glossary

AAQ: Acceptance and Action Questionnaire
ACT: Acceptance and commitment therapy
AHFMR: Alberta Heritage Foundation for Medical Research
AHP: Ambiguous Hallmark Programme
AI: Artificial Intelligence
AJOL: African Journals Online
ARS: Anger Rumination Scale
ART: Antiretroviral therapy/treatment
BDI: Beck Depression Inventory
BHI: Beck Hopelessness Inventory
CBT: Cognitive behaviour therapy
CDRS-R: Children’s Depression Rating Scale Revised
CENTRAL: Cochrane Central Register of Controlled Trials
CGAS: Children’s Global Assessment Scale
CGI-S: Clinician’s Global Impression Scale-Symptom Severity Subscale
CMT: Compassionate mind training
COVID-19: Corona virus 2019
CSQ: Client Satisfaction Questionnaire
CT: Cognitive therapy
DALY: Disability-adjusted life year
DAS: Dysfunctional Attitude Scale
DASS-42: Depression, Anxiety and Stress Scale-42
DISC-12: 12-item Discrimination and Stigma Scale
DSM: Diagnostic and Statistical Manual of Mental Disorders
EBM: Evidence-Based Medicine
EFT: Emotional freedom technique
EPA: European Congress of Psychiatry
EPDS: Edinburgh Postnatal Depression Scale
ESCAP: European Society for Child and Adolescent Psychiatry
ERQ-R: Emotion Regulation Questionnaire-Reappraisal Subscale
FFMQ: Five Facet Mindfulness Questionnaire
GAD-7: Generalised Anxiety Disorder-7
GAF-ID: Guided Act and Feel-Indonesia
GIM: Global Index Medicus
GPST: Group problem solving therapy
HARS: Hamilton Anxiety Rating Scale
HCW: Healthcare worker
HDRS: Hamilton Depression Rating Scale
HIC: High income country
HIV: Human immunodeficiency virus
HRSD-R: Hamilton Rating Scale for Depression-Revised
IACAPAP: International Association for Child and Adolescent Psychiatry and Allied Professionals
ICBT: Internet-based CBT
ICD: International Classification of Diseases
IPT: Interpersonal psychotherapy
ISI: Insomnia Severity Index
ITR: Individualised treatment rule
KAP: Knowledge, Attitude and Practises
KRISP: KwaZulu-Natal Research Innovation and Sequencing Platform
LMIC: Low- or middle-income country
MBCT: Mindfulness-based cognitive therapy
MDD: Major Depressive Disorder
MFAS: Maternal-Foetal Attachment Scale
mhGAP-IG: Mental Health Gap Action Plan Intervention Guide
MINI-KID: Mini International Neuropsychiatric Interview for Children and Adolescents
MSPSS: Multidimensional Scale of Perceived Social Support
MSRT: Ministry of Science, Research and Technology
N/A: not applicable
NCAST: Nursing Child Assessment Satellite Training
NCT: Narrative cognitive therapy
NIH: National Institutes of Health
(N)MA: (Network) meta-analysis
NR: not reported
PAAS: PREMIUM Abbreviated Activation Scale
PANSS: Positive Negative Syndrome Scale
PHQ-9: Patient Health Questionnaire-9
PHQ-ADS: Patient Health Questionnaire-Anxiety and Depression Scale
PRISMA: Preferred Reporting Items for Systematic Literature Reviews and Meta-Analysis
PRIME: PRogramme for Improving Mental health carE
PROSPERO: Prospective Register of Systematic Reviews
PSP: Personal Social Performance
PSQI: Pittsburgh Sleep Quality Index
PST: Problem solving therapy
QALY: Quality-adjusted life year
QoL: Quality of life
RCT: Randomised controlled trial
(R)REBT: (Religious) rational emotive behaviour therapy
SCCS: Self-Consistency and Congruence Scale
SCID-5: Structured clinical interview for DSM-V
SCS: Self-Compassion Scale
SDG: Sustainable Development Goals
SLR: Systematic literature review
SMS: Short messaging service
SPWB: Scales of Psychological Well-Being
SSI: Scale for Suicidal Ideation
UK: United Kingdom
UN: United Nation
UNICEF: United Nations Children’s Fund
US: United States
UWONET: Uganda Women’s Network
VAS: Visual analogue scale
WHO: World Health Organisation
WHO-5: World Health Organisation-Five Well-Being Index
WHO-ICF: World Health Organisation International Classification of Functioning, Disability and Health
WHOQoL-BREF: World Health Organisation Quality of Life Brief Version
YLD: Years lived with disability
