# Supplementary material for: Public health interventions to improve the mental health and mental health literacy of young people in resource-limited settings: a systematic review
Source: Front Public Health. 2026 Jun 2;14:1741504. doi: 10.3389/fpubh.2026.1741504 (PMC13269096; doi:10.3389/fpubh.2026.1741504)
Supplement: Supplementary file 1 [file Data_Sheet_1.DOCX]

Supplementary Material

# Supplementary Tables

Supplementary Table 1. Eligibility Criteria

| **Domain** | **Inclusion** | **Exclusion** |
| --- | --- | --- |
| **Population^a^** | - Young people aged 15–29 years with MDD and schizophrenia^b^ | - Paediatric population or older adults - People without severe mental illness |
| **Intervention** | Public health interventions (including in educational settings), including:^c^   - Self-care (e.g. social support/network, self-efficacy, self-help groups, self-help guides, support companions, seeking help, peer-to-peer programmes) - Patient care planning and patient care management (e.g. youth programmes, youth management, management plans, patient programmes) - Health education and patient education (e.g. caregiver advice, patient booklets, brochures, pamphlets, leaflets, learning tools) - Telemedicine (e.g. hotlines, telehealth, e-health, tele consultation, digital assistance, artificial intelligence [AI]) - Health services (e.g. community health services, mental health centres, rehabilitation facilities, mental health providers) - Collaborative care (e.g. integrated healthcare systems, interdisciplinary care services, multidisciplinary approach) - Behaviour therapy (e.g. cognitive behavioural therapy, counselling, mindfulness, meditation, holistic approaches) | - Any other interventions (e.g. pharmacological interventions) |
| **Comparator** | - Any or none | - N/A |
| **Outcomes** | - Characteristics of the intervention - Target population for the intervention - Time required to implement intervention and follow-up period - Assessments of the outcome of an intervention, including evaluating potential clinical benefits   - Quantitative impact assessment via formal, validated tools (e.g. WHO-Five Well-Being Index [WHO-5], Patient Health Questionnaire-9 [PHQ-9], Patient Health Questionnaire Anxiety and Depression Scale [PHQ-ADS], Beck Depression Inventory [BDI] etc.)   - Qualitative impact assessment (e.g. knowledge, attitudes and practices [KAP] survey) | - No relevant characteristics or outcomes reported |
| **Study design** | - Interventional studies e.g. RCTs and non-randomised interventional studies - Observational and real-world studies - SLR/(N)MAs^d^ | - Case reports |
| **Language** | - Any language^e^ | - N/A |
| **Publication type** | - Any publication type reporting novel data | - Narrative reviews or other publications not reporting novel data |
| **Other considerations** | - Full texts published in or since 2010^f^ - Congress abstracts published in or since 2022   Countries:   - Priority 1: studies conducted in Brazil, India, Kenya - Priority 2: studies conducted in LMICs and resource-limited high-income country [HIC] settings^g^ | - Interventions/programmes initiated before 2010 - Data collected before 2010 |

**Footnotes:** ^a^Populations included subgroups of patients with a specific condition or comorbidity (e.g. corona virus 2019 [COVID-19], HIV). ^b^Based on studies including patients with depression/schizophrenia reported as “severe” or “major”, or who met cut-offs for a validated tool such as the PHQ-9. A threshold of ≥80% of participants meeting the inclusion criteria was used (in alignment with most evidence review guidelines) to determine study inclusion. ^C^The list of public health interventions of interest was as defined in the Search Terms. ^d^SLR/(N)MAs were included at the title/abstract review stage for reference list searching. ^e^Articles from any language were tagged, included and underwent machine translation during the review process. ^f^The date limit of 2010 was specified as it was the year where the WHO published a Youth Mental Health declaration, aiming to define tangible and measurable targets for strengthening mental health services for young people in the next 10 years.(1) ^g^The list of countries classified as LMICs was defined as per the most recent World Bank classifications of low-income, lower-middle income and upper-middle-income countries.(2) Despite being categorised as priority 2, studies conducted in these countries were included in the review due to the limited volume of evidence from studies conducted in priority 1 countries.
**Abbreviations:** AI: artificial intelligence; BDI: Beck’s Depression Inventory; COVID-19: corona virus 2019; HIC: high-income country; HIV: human immunodeficiency virus; KAP: Knowledge, Attitude and Practices; LMIC: low- and middle-income country; MDD: major depressive disorder; N/A: not applicable; (N)MA: (network) meta-analysis; PHQ-9: Patient Health Questionnaire-Nine; PHQ-ADS: Patient Health Questionnaire Anxiety and Depression; RCT: randomised controlled trial; SLR: systematic literature review; WHO: World Health Organization; WHO-5: World Health Organization-Five Well-Being Index.

Supplementary Table 2. Search terms for MEDLINE, Embase and CENTRAL (searched simultaneously via the Ovid SP platform)

| **Term Group** | **#** | **Searches** |
| --- | --- | --- |
| **Young people** |  | Adolescent/ or Young Adult/ or Students/ or student/ or Child/ |
|  |  | (Adolescen$ or child$ or teen$ or youth$ or minor$ or student$ or juvenile$ or boy$ or girl$ or pediatric$ or paediatric$ or school$ or universit$ or (young adj (people$ or person$ or adult$))).ti,ab,kf,kw. |
|  |  | 1 or 2 |
| **Severe mental health** |  | exp Schizophrenia/ or Schizophrenia, Childhood/ or childhood schizophrenia/ or "Schizophrenia Spectrum and Other Psychotic Disorders"/ or schizophrenia spectrum disorder/ or Delusions/ or delusion/ or Hallucinations/ or hallucination/ |
|  |  | (delusion$ or hallucinat$ or hebephreni$ or oligophreni$ or paranoi$ or psychotic$ or psychosis or psychoses or schizo$).ti,ab,kf,kw. |
|  |  | Depressive Disorder, Major/ or major depression/ or *Depressive Disorder/ or *depression/ |
|  |  | (major depressi$ or clinical depressi$ or unipolar depressi$ or unipolar disorder or persistent depressive disorder or dysthymia).ti,ab,kf,kw. |
|  |  | or/4-7 |
| **LMICs and resource-limited settings** |  | (resource limited setting or Developing Countries).sh,kf,kw. |
|  |  | Resource-limited setting/ |
|  |  | (Africa or Asia or Caribbean or West Indies or South America or Latin America or Central America).hw,kf,ti,ab,cp,kw. |
|  |  | (Afghanistan or Albania or Algeria or Angola or Anguilla or Antigua or Barbuda or Argentina or Armenia or Azerbaijan or Azeri or Bangladesh or Barbados or Benin or Byelarus or Byelorussian or Belarus or Belorussia or Belize or Bhutan or Bolivia or Bosnia or Herzegovina or Hercegovin or Botswana or Botsuana or Motswana or Batswana or Brasil or Brazil or Burkina Faso or Burkina Fasso or Burkina or Burundi or Urundi or Cambodia or Cameroon or Cameron or Cape Verd or Cabo Verde or Central African Republic or Chad or Tchad or Chile or China or Chinese or Colombia or Columbia or Comoros or Comoro Islands or Comores or Comoran or Mayotte or Congo or Costa Rica or Cote d'Ivoire or Ivory Coast or Ivorian or Cook Islands or Cuba or Croat or Djibouti or Dominica or East Timor or East Timur or Timor Leste or Timorese or Ecuador or Equador or Egypt or El Salvador or Salvadoran or Eritrea or Ethiopia or Fiji or Gabon or Gambia or Gaza or Georgia Republic or Georgian or Abkhazia or Abchasia or South Ossetia or Ghana or Grenada or Grenadian or Guatemala or Guinea or Guinea Bissau or Guian or Guyana or Haiti or Honduras or India or Indian or Indonesia or Iran or Iraq or Jamaica or Jordan or Kazakhstan or Kazakh or Kenya or Kiribati or Korea or Kosovo or Kosova or Kyrgyzstan or Kirghizia or Kyrgyz or Kirghiz or Kirgizstan or Lao PDR or Laos or Laotian or Lebanon or Lebanese or Lesotho or Mosotho or Basotho or Liberia or Libya or Macedonia or FYROM or Madagascar or Malagasy or Malaysia or Malaya or Malay or Sabah or Sarawak or Malawi or Maldives or Maldivan or Mali or Malian or Marshall Islands or Marshallese or Mauritania or Mauritius or "Agalega Islands" or Mexico or Mexican or Micronesia or "Middle East" or Moldova or Moldovia or Transnistria or Mongolia or Montenegro or Montserrat or Morocco or Moroccan or Mozambique or Mozambican or Myanmar or Myanma or Burma or Burmese or Namibia or Nauru or Niue or Nepal or Nicaragua or Niger or Nigerien or Nigeria or Oman or Pakistan or Palau or Palestine or Palestinian or Panama or Panamanian or Paraguay or Papua New Guinea or Peru or Peruvian or Philippines or Philipines or Phillipines or Phillippines or Filipino or Philipino or Philippino or Phillipino or Phillippino or Rwanda or Ruanda or "Saint Helen" or "St Helen" or "Saint Kitts" or "St Kitts" or Kittian or Nevis or "Saint Lucia" or "St Lucia" or "Saint Vincent" or "St Vincent" or Vicentian or Grenadines or Samoa or Sao Tome or Senegal or Serbia or Seychelles or "Sierra Leone" or "Sri Lanka" or Ceylon or "Solomon Island" or Somali or "South Africa" or Sudan or Sudanese or Surinam or Swaziland or Swazi or Eswatini or Syria or Syrian or Tajikistan or Tadzhikistan or Tadjikistan or Tajik or Tadzhik or Tanzania or Thailand or Thai or Togo or Togolese or Tonga or Tunisia or Tokelau or Trinidad or Tobago or Turkey or Turkish or Turks or Turkmenistan or Turkmen or Tuvalu or Uganda or Ukraine or Ukrainian or Uruguay or Uzbekistan or Uzbek or Vanuatu or Venezuela or Vietnam or Viet Nam or Wallis Futuna or West Bank or Yemen or Zambia or Zimbabwe).hw,kf,ti,ab,cp,kw. |
|  |  | ((developing or less$ developed or under developed or underdeveloped or middle income or low$ income or underserved or under served or deprived or poor$ or resource limited) adj (countr$ or nation? or population? or world or setting?)).ti,ab. |
|  |  | ((developing or less$ developed or under developed or underdeveloped or middle income or low$ income) adj (economy or economies)).ti,ab. |
|  |  | (low$ adj (gdp or gnp or gross domestic or gross national)).ti,ab. |
|  |  | (low adj3 middle adj3 countr$).ti,ab. |
|  |  | (lmic or lmics or third world or lami countr$).ti,ab. |
|  |  | transitional countr$.ti,ab. |
|  |  | or/9-18 |
| **Interventional studies** |  | Randomized Controlled Trials as Topic/ or "randomized controlled trial (topic)"/ |
|  |  | Randomized Controlled Trial/ |
|  |  | Random Allocation/ or randomization/ |
|  |  | Double-Blind Method/ or double blind procedure/ |
|  |  | Single-Blind Method/ or single blind procedure/ |
|  |  | Cross-Over Studies/ or crossover procedure/ |
|  |  | Placebos or placebo/ |
|  |  | exp Clinical Trials as Topic/ or exp "clinical trial (topic)"/ |
|  |  | Clinical Trial/ |
|  |  | Clinical Trial, Phase II/ or phase 2 clinical trial/ or Clinical Trial, Phase III/ or phase 3 clinical trial/ or Clinical Trial, Phase IV/ or phase 4 clinical trial/ |
|  |  | Controlled Clinical Trial/ or Adaptive Clinical Trial/ or multicenter study/ |
|  |  | randomized controlled trial.pt. |
|  |  | clinical trial.pt. |
|  |  | (clinical trial, phase ii or clinical trial, phase iii or clinical trial, phase iv).pt. |
|  |  | (controlled clinical trial or multicenter study).pt. |
|  |  | (clinical adj trial$).ti,ab,kf,kw. |
|  |  | ((singl$ or doubl$ or treb$ or tripl$) adj (blind$3 or mask$3)).ti,ab,kf,kw. |
|  |  | placebo$.ti,ab,kf,kw. |
|  |  | (allocat$ adj2 random$).ti,ab,kf,kw. |
|  |  | Randomi?ed adj2 trial$.ti,ab,kf,kw. |
|  |  | rct.ti,ab,kf. |
|  |  | (single arm adj3 (trial$ or stud$)).ti,ab,kf,kw. |
|  |  | (open label adj (trial$ or stud$)).ti,ab,kf,kw. |
|  |  | (non blinded adj (trial$ or stud$)).ti,ab,kf,kw. |
|  |  | (pragmatic trial$ or pragmatic stud$).ti,ab,kf,kw. or Pragmatic Clinical Trial/ or pragmatic trial/ |
|  |  | or/20-44 |
| **Observational and real-world studies** |  | Epidemiologic Studies/ |
|  |  | Observational Study/ |
|  |  | Cohort Studies/ or cohort analysis/ |
|  |  | exp Case-Control Studies/ or exp case control study/ |
|  |  | Cross-Sectional Studies/ or cross-sectional study/ |
|  |  | Clinical Study/ |
|  |  | Follow-Up Studies/ or follow up/ |
|  |  | Longitudinal Studies/ or longitudinal study/ |
|  |  | Retrospective Studies/ or retrospective study/ |
|  |  | (Prospective Studies/ not Randomized Controlled Trials as Topic/) or (prospective study/ not "randomized controlled trial (topic)"/) |
|  |  | (observational adj (study or studies)).ti,ab,kf,kw. |
|  |  | (cohort adj (study or studies)).ti,ab,kf,kw. |
|  |  | cohort analy$.ti,ab,kf,kw. |
|  |  | case control.ti,ab,kf,kw. |
|  |  | cross sectional.ti,ab,kf,kw. |
|  |  | (follow up adj (study or studies)).ti,ab,kf. |
|  |  | longitudinal.ti,ab,kf,kw. |
|  |  | retrospective.ti,ab,kf,kw. |
|  |  | (chart adj3 review$).ti,ab,kf,kw. |
|  |  | exp Registries/ or exp disease registry/ |
|  |  | (registry or registries).ti,ab,kf,kw. |
|  |  | or/46-66 |
| **Intervention programme** |  | exp Self Care/ or Self Efficacy/ or self concept/ or Social Support/ or Self-Help Groups/ or self help/ |
|  |  | (((self or selves or personal$ or themsel$) adj2 (assess$ or care$ or caring or control$ or efficacy$ or effectiv$ or guid$ or help$ or intervention$ or manag$ or pace$ or therap$ or treat$)) or ((network$ or support$ or therap$ or treat$) adj2 (club$ or group$ or social$ or peer$ or friend$ or companion$ or buddy))).ti,ab,kf,kw. |
|  |  | (help$ adj2 seek$).ti,ab,kf,kw. |
|  |  | Patient Care Planning/ or Case Management/ or Patient Care Management/ or *patient care/ |
|  |  | (((action$ or care$ or manag$ or individual$ or patient$ or youth$ or young$ or child$ or self or personal$) adj2 (plan$ or program$)) or (goal$ adj2 (care$ or set$)) or (case$ adj2 manag$)).ti,ab,kf,kw. |
|  |  | Health Education/ or Patient Education as Topic/ or *patient education/ or Patient Education Handout/ or Pamphlets/ or exp Health Promotion/ or Information Centers/ or information center/ or Information Services/ or information service/ or Information Dissemination/ or Libraries/ or library/ or Library Services/ or Health Literacy/ |
|  |  | ((health adj4 (consumer$ or educat$ or promot$ or literacy or train$)) or ((patient$ or carer$ or caregiver$ or care-giver$ or consumer$ or young$ or youth$ or child$) adj2 (advis$ or advice$ or counsel$ or booklet$ or brochure$ or communicat$ or dvd$ or educat$ or forum$ or handout$ or hand-out$ or informat$ or leaflet$ or learn$ or lesson$ or material$ or pamphlet$ or promot$ or resource$ or support$ or teach$ or tool$ or train$ or video$ or website$))).ti,ab,kf,kw. |
|  |  | Telemedicine/ or Computers/ or computer/ or exp Computers, Handheld/ or personal digital assistant/ or exp Internet/ or Mobile Applications/ or mobile application/ or Social Networking/ or social network/ or Electronic Mail/ or e-mail/ or Text Messaging/ or Telephone/ or exp Cell Phones/ or mobile phone/ or Hotlines/ or hotline/ or exp Teaching Materials/ or *teaching/ or Computer-Assisted Instruction/ or Videoconferencing/ |
|  |  | ((digihealth$ or digi-health$ or digital$ or mobile$ or tele$ or ehealth or e-health or etherap$ or e-therap$ or etreat$ or e-treat$ or mhealth$ or m-health$) or ((online or internet or remote or phone$ or telephone$) adj2 (care$ or consult$ or manage$ or monitor$ or therap$ or treatment$)) or (android$ or blog$ or facebook$ or facetime$ or face time$ or helpline$ or hotline$ or ipad$ or iphone$ or mobile phone$ or cell phone$ or personal digital assistant$ or mp3$ or podcast$ or skype$ or smartphone$ or smart-phone$ or social media$ or social network$ or sms or text messag$ or twitter$ or tweet$ or tutorial$ or wiki$ or youtube$ or artific$ intelligen$) or ((digital$ or mobile$ or phone$ or tablet$ or portable$) adj4 app$)).ti,ab,kf,kw. |
|  |  | Health Services/ or health service/ or exp Community Health Services/ or community care/ or exp Community Psychiatry/ or social psychiatry/ or Custodial Care/ or Personal Health Services/ or Rehabilitation/ or community mental health center/ |
|  |  | ((communit$ or mental$ or custod$ or psychosocial$ or psycho social$ or reabl$ or rehabilitat$) adj3 (care$ or agenc$ or deliver$ or department$ or facilit$ or institution$ or network$ or organi?ation$ or provider$ or provision$ or partner$ or sector$ or service$ or setting$)).ti,ab. |
|  |  | exp "Delivery of Health Care, Integrated"/ or integrated health care system/ or Intersectoral Collaboration/ or Interinstitutional Relations/ or Interprofessional Relations/ or public relations/ or collaborative care team/ or multidisciplinary team/ |
|  |  | (((integrat$ or collaborat$ or colocat$ or co locat$ or cross sector$ or interagenc$ or inter agenc$ or interdisciplin$ or inter disciplin$ or interinstitution$ or inter institution$ or interorgani?$ or inter organi?$ or intersector$ or inter sector$ or intraprofession$ or intra profession$ or joined up or joint or merged or multiagenc$ or multi agenc$ or multidisciplin$ or multi disciplin$ or multiprofession$ or multi profession$ or multisector$ or multi sector$ or overlap$ or share$ or sharing$ or pool$ or transdisciplin$ or trans disciplin$) adj3 (access$ or assess$ or care$ or communit$ or consult$ or model$ or program$ or review$ or service$ or staff$ or system$ or team$ or transfer$ or work$ or budget$ or financ$ or fund$ or payment$ or resource$ or educat$ or develop$ or approach$ or train$)) or task$ shift$ or decentrali?$ or de centrali?$).ti,ab,kf,kw. |
|  |  | Behavior Therapy/ or Cognitive Behavioral Therapy/ or Counseling/ or Directive Counseling/ or Child Guidance/ or Psychology, Adolescent/ or Psychology, Child/ or child psychology/ or Motivational Interviewing/ or Mindfulness/ or Meditation/ or Yoga/ |
|  |  | (counsel$ or advice$ or advise$ or advisor$ or pyschotherap$ or psycho therap$ or CBT$ or cCBT or CBASP or ((behaviour$ or behavior$ or cognit$) adj3 (technique$ or therap$ or chang$ or modif$ or support$ or intervention$ or session$ or program$ or workshop$)) or motivat$ interview$ or life skill$ or mindful$ or meditat$ or yoga or holistic$).ti,ab,kf,kw. |
|  |  | or/68-82 |
| **Exclusions** |  | conference$.pt. |
|  |  | limit 84 to yr="1974-2021" |
|  |  | exp animals/ not exp humans/ |
|  |  | (comment or editorial or case reports or historical article).pt. |
|  |  | editorial/ or case report/ |
|  |  | (case stud$ or case report$).ti. |
|  |  | or/85-89 |
| **Combination (MEDLINE and Embase)** |  | 3 and 8 and 19 and (45 or 67) and 83 |
|  |  | 91 not 90 |
| **Combination (CENTRAL)** |  | 3 and 8 and 19 and 83 |
| **MEDLINE** |  | 92 use ppez |
| **Embase** |  | 92 use oemezd |
| **CENTRAL** |  | 93 use cctz |
|  |  | 96 not 84 |
| **Total** |  | 94 or 95 or 97 |
|  |  | Limit 98 to yr="2010-current" |
|  |  | **Remove duplicates from 99** |

**Footnote:** Searches ran on 5^th^ July 2024.
**Databases:** Evidence-Based Medicine (EBM) Reviews - Cochrane Central Register of Controlled Trials June 2024, Embase 1974 to 2024 July 03, Ovid MEDLINE(R) Epub Ahead of Print and In-Process, In-Data-Review & Other Non-Indexed Citations and Daily July 03, 2024.
**Abbreviations:** CENTRAL: Cochrane Central Register of Controlled Trials; EBM: Evidence-Based Medicine; LMIC: low- and middle-income country.

Supplementary Table 3. Search terms for PsycINFO (searched via the APA PsycNet platform)

| **Term Group** | **#** | **Search Logic** | **Terms** | **Search in** |
| --- | --- | --- | --- | --- |
| **Population** |  | AND | adolescen* or child* or teen* or youth* or minor* or student* or juvenile* or boy* or girl* or pediatric* or paediatric* or school* or universit* or young people or young person or young adult | Abstract |
|  |  |  | delusion* or hallucinat* or hebephreni* or oligophreni* or paranoi* or psychotic* or psychosis* or psychoses* or schizo* or major depressi* or clinical depressi* or unipolar depressi* or unipolar disorder or persistent depressive disorder or dysthymia | Abstract |
| **Country** |  | OR | Afghanistan or Albania or Algeria or Angola or Anguilla or Antigua or Barbuda or Argentina or Armenia or Azerbaijan or Azeri or Bangladesh or Barbados or Benin or Byelarus or Byelorussian or Belarus or Belorussia or Belize or Bhutan or Bolivia or Bosnia or Herzegovina or Hercegovin or Botswana or Botsuana or Motswana or Batswana or Brasil or Brazil or Burkina Faso or Burkina Fasso or Burkina or Burundi or Urundi or Cambodia or Cameroon or Cameron or Cape Verd or Cabo Verde or Central African Republic or Chad or Tchad or Chile or China or Chinese or Colombia or Columbia or Comoros or Comoro Islands or Comores or Comoran or Mayotte or Congo or Costa Rica or Cote d'Ivoire or Ivory Coast or Ivorian or Cook Islands or Cuba or Croat or Djibouti or Dominica or East Timor or East Timur or Timor Leste or Timorese or Ecuador or Equador or Egypt or El Salvador or Salvadoran or Eritrea or Ethiopia or Fiji or Gabon or Gambia or Gaza or Georgia Republic or Georgian or Abkhazia or Abchasia or South Ossetia or Ghana or Grenada or Grenadian or Guatemala or Guinea or Guinea Bissau or Guian or Guyana or Haiti or Honduras or India or Indian or Indonesia or Iran or Iraq or Jamaica or Jordan or Kazakhstan or Kazakh or Kenya or Kiribati or Korea or Kosovo or Kosova or Kyrgyzstan or Kirghizia or Kyrgyz or Kirghiz or Kirgizstan or Lao PDR or Laos or Laotian or Lebanon or Lebanese or Lesotho or Mosotho or Basotho or Liberia or Libya or Macedonia or FYROM or Madagascar or Malagasy or Malaysia or Malaya or Malay or Sabah or Sarawak or Malawi or Maldives or Maldivan or Mali or Malian or Marshall Islands or Marshallese or Mauritania or Mauritius or Agalega Islands or Mexico or Mexican or Micronesia or Middle East or Moldova or Moldovia or Transnistria or Mongolia or Montenegro or Montserrat or Morocco or Moroccan or Mozambique or Mozambican or Myanmar or Myanma or Burma or Burmese or Namibia or Nauru or Niue or Nepal or Nicaragua or Niger or Nigerien or Nigeria or Oman or Pakistan or Palau or Palestine or Palestinian or Panama or Panamanian or Paraguay or Papua New Guinea or Peru or Peruvian or Philippines or Philipines or Phillipines or Phillippines or Filipino or Philipino or Philippino or Phillipino or Phillippino or Rwanda or Ruanda or Saint Helen or St Helen or Saint Kitts or St Kitts or Kittian or Nevis or Saint Lucia or St Lucia or Saint Vincent or St Vincent or Vicentian or Grenadines or Samoa or Sao Tome or Senegal or Serbia or Seychelles or Sierra Leone or Sri Lanka or Ceylon or Solomon Island or Somali or South Africa or Sudan or Sudanese or Surinam or Swaziland or Swazi or Eswatini or Syria or Syrian or Tajikistan or Tadzhikistan or Tadjikistan or Tajik or Tadzhik or Tanzania or Thailand or Thai or Togo or Togolese or Tonga or Tunisia or Tokelau or Trinidad or Tobago or Turkey or Turkish or Turks or Turkmenistan or Turkmen or Tuvalu or Uganda or Ukraine or Ukrainian or Uruguay or Uzbekistan or Uzbek or Vanuatu or Venezuela or Vietnam or Viet Nam or Wallis Futuna or West Bank or Yemen or Zambia or Zimbabwe | Geographic Location |
|  |  |  | (developing or “less* developed” or “under developed” or underdeveloped or “middle income” or “low* income” or underserved or “under served” or deprived or “resource limited” or “resource-limited”) NEAR/1 (countr* or nation* or population* or setting*) | Abstract |
| **Intervention** |  | - | ((((self or selves or personal* or themsel*) near/2 (assess* or care* or caring or control* or efficacy* or effectiv* or guid* or help* or intervention* or manag* or pace* or therap* or treat*)) or ((network* or support* or therap* or treat*) near/2 (club* or group* or social* or peer* or friend* or companion* or buddy*)) or (help* near/2 seek*) or ((action* or care* or manag* or individual* or patient* or youth* or young* or child* or self or personal*) near/2 (plan* or program*)) or ((goal* near/2 (care* or set*) or (case* near/2 manag*)) or (health near/4 (consumer* or educat* or promot* or literacy or train*)) or ((patient* or carer* or caregiver* or care-giver* or consumer* or young* or youth* or child*) near/2 (advis* or advice* or counsel* or booklet* or brochure* or communicat* or dvd* or educat* or forum* or handout* or hand-out* or informat* or leaflet* or learn* or lesson* or material* or pamphlet* or promot* or resouce* or support* or teach* or tool* or train* or video* or website*)) or (digihealth* or digi-health* or digital* or mobile* or tele* or ehealth or e-health or etherap* or e-therap* or etreat* or e-treat* or mhealth* or m-health*) or ((online or internet or remote or phone* or telephone*) near/2 (care* or consult* or manage* or monitor* or therap* or treatment*)) or (android* or blog* or facebook* or facetime* or face time* or helpline* or hotline* or ipad* or iphone* or mobile phone* or cell phone* or personal digital assistant* or mp3* or podcast* or skype* or smartphone* or smart-phone* or social media* or social network* or sms or text messag* or twitter* or tweet* or tutorial* or wiki* or youtube* or artific* intelligen*) or ((digital* or mobile* or phone* or tablet* or portable*) near/4 app*) or (communit* or mental* or custod* or psychosocial* or psycho social* or reabl* or rehabilitat*) near/3 (care* or agenc* or deliver* or department* or facilit* or institution* or network* or organi?ation* or provider* or provision* or partner* or sector* or service* or setting* or base*) or ((integrat* or collaborat* or colocat* or co locat* or cross sector* or interagenc* or inter agenc* or interdisciplin* or inter disciplin* or interinstitution* or inter institution* or interorgani?* or inter organi?* or intersector* or inter sector* or intraprofession* or intra profession* or joined up or joint or merged or multiprofession* or multi profession* or multisector* or multi sector* or overlap* or share* or sharing* or pool* or transdisciplin* or trans disciplin*) near/3 (access* or assess* or care* or communit* or consult* or model* or program* or review* or service* or staff* or system* or team* or transfer* or work* or budget* or financ* or fund* or payment* or resource* or educat* or develop* or approach* or train*)) or task* shift* or decentrali?* or de centrali?* or counsel* or advice* or advise* or advisor* or psuchotherap* or psycho therap* or CBT* or cCBT or CBASP or ((behaviour* or behavior* or cognit*) near/3 (technique* or therap* or chang* or modif* or support* or intervention* or session* or program* or workshop*)) or motivat* interview* or life skill* or mindful* or meditat* or yoga or holistic*)) | Abstract |
| ***Combination*** |  | AND | Combined Total | - |
| ***Filters*** | - | - | **Date**: 2010 to 2024 | - |
|  |  |  | **Document** **Type**: Journal Article | - |
|  |  |  | **Age** **Group**: Adolescence; Young Adulthood | - |
| **Total** | - | - | **Total including filters** | - |

**Footnote:** Searches ran on 3^rd^ July 2024.
**Databases:** PsycINFO 1967 to July 03 2024.

Supplementary Table 4. Search strategies for websites

| **Website** | **Search Strategy** |
| --- | --- |
| [WHO](https://www.who.int/) | Search the following in Google, with date limit 2010 to now and screen first 50 hits:   - (adolescent OR teen* OR youth OR "young person" OR "young adult" OR student OR school OR university) AND (schizophrenia OR schizophrenic OR hebephrenia OR hebephrenic OR psychosis OR psychotic OR depressed OR depressive OR depression OR dysthymia OR dysthymic) site:https://www.who.int/ after:2009 |
| [Being Initiative](https://being-initiative.org/) | Search the following in Google:   - (schizophrenia OR schizophrenic OR hebephrenia OR hebephrenic OR psychosis OR psychotic OR depressed OR depressive OR depression OR dysthymia OR dysthymic) site:https://being-initiative.org/ |
| [Global Coalition for Youth Mental Health](https://www.youthmentalhealthcoalition.org/) | Search the following in Google:   - (schizophrenia OR schizophrenic OR hebephrenia OR hebephrenic OR psychosis OR psychotic OR depressed OR depressive OR depression OR dysthymia OR dysthymic) site:https://www.youthmentalhealthcoalition.org/ |
| [Orygen](https://www.orygen.org.au/) | Search the following in Google, with date limit 2010 to now and screen first 50 hits:   - (adolescent OR teen* OR youth OR "young person" OR "young adult" OR student OR school OR university) AND (schizophrenia OR schizophrenic OR hebephrenia OR hebephrenic OR psychosis OR psychotic OR depressed OR depressive OR depression OR dysthymia OR dysthymic) site:https://www.orygen.org.au/ after:2009 |
| [Verywell Mind](https://www.verywellmind.com/) | Search the following in Google, with date limit 2010 to now and screen first 50 hits:   - (adolescent OR teen* OR youth OR "young person" OR "young adult" OR student OR school OR university) AND (schizophrenia OR schizophrenic OR hebephrenia OR hebephrenic OR psychosis OR psychotic OR depressed OR depressive OR depression OR dysthymia OR dysthymic) site:https://www.verywellmind.com/ after:2009 |

**Footnote:** Searches ran on 2^nd^ October 2024. **Abbreviations:** WHO: World Health Organization.

Supplementary Table 5. Search strategies for non-English databases

| **Source** | **Search Strategy** |
| --- | --- |
| [GIM](https://www.globalindexmedicus.net/) | (ab:((adolescen* OR teen* OR youth* OR minor* OR student* OR pupil* OR school* OR universit*) OR (young AND people* OR person* or adult*))) |
|  | ab:(((ab:(delusion* OR hallucinat* OR hebephreni* OR oligophreni* OR paranoi* OR psychotic* OR psychosis OR psychoses OR schizo*)) OR (ab:(major depressi* OR clinical depressi* OR unipolar depressi* OR unipolar disorder OR persistent depressive disorder OR dysthymia)))) |
|  | Combination of both |
|  | AND type_of_study:("qualitative_research" OR "prognostic_studies" OR "risk_factors_studies" OR "observational_studies" OR "diagnostic_studies" OR "screening_studies" OR "clinical_trials" OR "evaluation_studies") |
|  | AND (year_cluster:[2019 TO 2024]) |
| [AJOL](https://www.ajol.info/index.php/ajol) | (adolescent OR teen OR youth OR student OR school OR university OR young) AND ("schizophrenia" OR "schizo*"OR "major depressive" OR "major depression") AND (intervention OR "health service" OR campaign OR program OR trial OR initiative or promotion) site:ajol.info  Then click on “Tools” and apply a date limit from 2019 |

**Footnote:** Searches ran on GIM on 30^th^ September 2024. Searches ran on AJOL on 27^th^ September 2024.  **Abbreviations:** AJOL: African Journals Online; GIM: Global Index Medicus.

Supplementary Table 6. Search strategies for Congress Proceedings

| **Conference** | **Link** | **Search Strategy** |
| --- | --- | --- |
| European Congress of Psychiatry (EPA)   - EPA 2023 - EPA 2022 | **2023:** <https://2023.epa-congress.org/abstracts/epa-2023-digital-abstract-flipbook/>  **2022:** <https://2022.epa-congress.org/epa-2022-digital-abstract-supplement/> | In Adobe, use Ctrl+F and click the cog icon and then ‘Open Full Acrobat Search’  Click 'Show More Options'   1. Under 'Return results containing', select 'Match Any of the words’ and **tick** checkbox indicating ‘Whole words only’  Type in the following search terms in one line and click search:  - aged ages   Click through and screen abstracts for an age range within our eligibility criteria, highlight the title for each eligible abstract   1. Under 'Return results containing', select 'Match Exact word or phrase’ and **untick** checkbox indicating ‘Whole words only’  Type in the following search terms and click search for each phrase separately:  - years old - mean age - median age - average age - young people   Click through and screen abstracts for an age range within our eligibility criteria, highlight the title for each eligible abstract |
| Karolinska Institutet – UNICEF Joint Conference on Global Child and Adolescent Mental Health 2024 | <https://brighterfutures2024.ki.se/> | In Adobe, use Ctrl+F and click the cog icon and then ‘Open Full Acrobat Search’  Click 'Show More Options'  (1) Under 'Return results containing', select 'Match Any of the words’ and **untick** checkbox indicating ‘Whole words only’  Type in the following search terms in one line and click search:   - schizophreni hebephreni psychosis psychoti dysthymi   Click through and screen abstracts based on our eligibility criteria, and highlight the titles of eligible abstracts  (2) Under 'Return results containing', select 'Match Exact word or phrase’ and **untick** checkbox indicating ‘Whole words only’   - major depress   Screen titles for relevance against the eligibility criteria |
| European Society for Child and Adolescent Psychiatry (ESCAP)   - ESCAP 2022 | <https://www.escap.eu/uploads/Events/Maastricht%202021/escap-2022-abstractbook-1.pdf> | In Adobe, use Ctrl+F and click the cog icon and then ‘Open Full Acrobat Search’  Click 'Show More Options'  (1) Under 'Return results containing', select 'Match Any of the words’ and **untick** checkbox indicating ‘Whole words only’  Type in the following search terms in one line and click search:   - schizophreni hebephreni psychosis psychoti dysthymi   Click through and screen abstracts based on our eligibility criteria, and highlight the titles of eligible abstracts  (2) Under 'Return results containing', select 'Match Exact word or phrase’ and **untick** checkbox indicating ‘Whole words only’   - major depress   Click through and screen abstracts based on our eligibility criteria, and highlight the titles of eligible abstracts |
| International Association for Child and Adolescent Psychiatry and Allied Professionals (IACAPAP) 2024 | <https://www.iacapap2024.com/ingles/trabalhos/aprovados.php#topo> | In the approved submissions page, Leave Title/Author empty and search through abstracts of each theme:   - Regional topics - General Child and Adolescent Mental Health - Psychiatric disorders and comorbid conditions - Principles of treatment and care - Technologies and mental health - Mental health and allied disciplines   Screen titles for relevance against the eligibility criteria |

**Footnote:** Searches were run between 23^rd^ and 26^th^ September 2024.
**Abbreviations:** EPA: European Congress of Psychiatry; ESCAP: European Society for Child and Adolescent Psychiatry; IACAPAP: International Association for Child and Adolescent Psychiatry and Allied Professionals; UNICEF: United Nations Children's Fund.

Supplementary Table 7. Items extracted from included publications

| **Category** | **Extracted items** |
| --- | --- |
| Study characteristics | - Study design - Geography - Study setting - Study period - Sources of funding - Key development partners |
| Intervention characteristics | - Intervention type - Intervention setting - Intervention aim - Intervention framework - Time required for implementation - Intervention description - Intervention development - Intervention duration - Follow-up period |
| Target population characteristics | - Age - Sex - Eligibility criteria - Type of severe mental illness - Population setting - Socioeconomic status - Population occupation - Educational level - Comorbidities |
| Intervention outcome assessment | - Intervention impact assessment using quantitative or qualitative tools at any timepoint, including: - Literacy - Knowledge - Attitudes - Mental health symptoms - Functioning |

Supplementary Table 8. Results of the quality assessment

| **Study name** | **Question/objective sufficiently described?** | **Study design evident and appropriate?** | **Method of subject/comparison group selection or source of information/input variables described and appropriate?** | **Subject (and comparison group, if applicable) characteristics sufficiently described?** | **If interventional and random allocation was possible, was it described?** | **If interventional and blinding of investigators was possible, was it reported?** | **If interventional and blinding of subjects was possible, was it reported?** | **Outcome and (if applicable) exposure measure(s) well defined and robust to measurement / misclassification bias? Means of assessment reported?** | **Sample size appropriate?** | **Analytic methods described/justified and appropriate?** | **Some estimate of variance is reported for the main results?** | **Controlled for confounding?** | **Results reported in sufficient detail?** | **Conclusions supported by the results?** | **Score** |
| --- | --- | --- | --- | --- | --- | --- | --- | --- | --- | --- | --- | --- | --- | --- | --- |
| **MDD** | | | | | | | | | | | | | | | |
| Alavi 2013 (3) | Y | Y | Y | Y | N/A | N/A | N/A | Y | Y | Y | Y | P | Y | Y | 0.95 |
| Alhusen 2021 (4) | Y | Y | Y | Y | Y | N | N | Y | Y | P | Y | P | Y | Y | 0.79 |
| Arjadi 2018 (5) | Y | Y | Y | Y | Y | Y | Y | Y | Y | Y | Y | Y | Y | Y | 1.00 |
| Bantjes 2021 (6) | Y | Y | P | Y | N/A | N/A | N/A | Y | Y | Y | Y | Y | Y | Y | 0.95 |
| Benjet 2023 (7) | Y | Y | Y | Y | Y | Y | N/A | Y | Y | Y | Y | Y | Y | Y | 1.00 |
| Chen 2024 (8) | Y | Y | N | P | P | N | N | P | Y | Y | Y | P | P | Y | 0.61 |
| Church 2012 (9) | Y | Y | Y | Y | P | Y | N | Y | Y | Y | Y | Y | Y | Y | 0.89 |
| Eseadi 2022 (10) | Y | Y | Y | Y | Y | Y | N | Y | Y | Y | Y | Y | Y | Y | 0.93 |
| Ezeudu 2020 (11) | Y | Y | P | Y | Y | P | P | Y | Y | Y | Y | Y | Y | Y | 0.89 |
| Far 2017 (12) | Y | Y | Y | Y | P | N | N | Y | P | Y | Y | Y | Y | Y | 0.79 |
| Fereydouni 2022 (13) | Y | Y | Y | Y | Y | N | N | Y | Y | Y | Y | Y | Y | Y | 0.86 |
| Gureje 2019 (14) | Y | Y | Y | Y | P | P | P | Y | Y | Y | Y | Y | Y | Y | 0.89 |
| Gureje 2022 (15) | Y | Y | Y | Y | P | Y | N/A | Y | Y | Y | Y | Y | Y | Y | 0.96 |
| Kaaya 2022 (16) | Y | Y | Y | Y | Y | N/A | N/A | Y | Y | Y | Y | Y | Y | Y | 1.00 |
| Kondradt 2018 (17) | Y | Y | Y | Y | N/A | Y | Y | Y | P | Y | Y | Y | Y | Y | 0.96 |
| Nakku 2021 (18) | Y | Y | Y | Y | N/A | N/A | N/A | Y | Y | Y | Y | N | Y | Y | 0.91 |
| Nejati 2019 (19) | Y | Y | Y | Y | P | Y | Y | Y | P | Y | Y | Y | Y | Y | 0.93 |
| Ofoegbu 2020 (20) | Y | Y | Y | Y | P | N | N | Y | Y | Y | Y | Y | N | Y | 0.75 |
| Olashore 2023 (21) | Y | Y | Y | Y | P | Y | N | Y | Y | Y | Y | P | Y | Y | 0.86 |
| Osborn 2020 (22) | Y | Y | P | P | Y | Y | N | Y | P | Y | Y | Y | P | Y | 0.79 |
| Savari 2021 (23) | Y | Y | Y | P | Y | N | N | Y | Y | Y | Y | P | Y | Y | 0.79 |
| Singla 2021 (24) | Y | Y | Y | Y | Y | Y | N | Y | Y | Y | Y | Y | Y | Y | 0.93 |
| Srivastava 2020 (25) | Y | Y | Y | Y | P | P | P | Y | Y | Y | Y | Y | Y | Y | 0.89 |
| Toth 2013 (26) | Y | Y | Y | Y | Y | Y | N/A | Y | Y | Y | Y | Y | Y | Y | 1.00 |
| Xu 2023 (27) | Y | Y | P | Y | P | Y | N/A | Y | Y | Y | Y | P | Y | Y | 0.88 |
| Zemestani 2020 (28) | Y | Y | P | Y | Y | N | N | Y | Y | Y | Y | Y | Y | Y | 0.82 |
| **Schizophrenia** | | | | | | | | | | | | | | | |
| Correa-Oliveira 2022 (29) | Y | Y | P | Y | N/A | N/A | N/A | P | Y | N | Y | N | Y | Y | 0.73 |
| Mlay 2022 (30) (study protocol) | Y | Y | Y | N/A | Y | N/A | N/A | Y | Y | Y | N/A | N | N/A | N/A | 0.88 |
| Ngoc 2016 (31) | Y | Y | Y | Y | P | Y | N/A | Y | Y | Y | Y | Y | Y | Y | 0.96 |
| She 2016 (32) | Y | Y | Y | Y | P | Y | N | Y | Y | Y | Y | Y | Y | Y | 0.89 |

**Key**: Y: criteria fully met by publication(s); P: criteria partially met by publication(s); N: criteria not met by publication(s); N/A: criteria not applicable to these publications.

# Supplementary Figures

Supplementary Figure 1. Summary of quality assessment

**Abbreviations:** NA: Not applicable.

# References

1. Coughlan H, Cannon M, Shiers D, Power P, Barry C, Bates T, et al. Towards a new paradigm of care: the International Declaration on Youth Mental Health. 2013.

2. The World Bank. World Bank Country and Lending Groups. Available at <https://datahelpdesk.worldbank.org/knowledgebase/articles/906519-world-bank-country-and-lending-groups> (accessed 29 November 2024) 2024 [

3. Alavi A, Sharifi B, Ghanizadeh A, Dehbozorgi G. Effectiveness of Cognitive-Behavioral Therapy in decreasing suicidal ideation and hopelessness of the adolescents with previous suicidal attempts. Iranian journal of pediatrics. 2013;Vol.23(4):467-72p.

4. Alhusen JL, Hayat, M. J., & Borg, L. A Pilot Study of a Group-Based Perinatal Depression Intervention on Reducing Depressive Symptoms and Improving Maternal-Fetal Attachment and Maternal Sensitivity. Arch Womens Ment Health. 2021;24(1):145–54.

5. Arjadi R, Nauta MH, Scholte WF, Hollon SD, Chowdhary N, Suryani AO, et al. Internet-based behavioural activation with lay counsellor support versus online minimal psychoeducation without support for treatment of depression: a randomised controlled trial in Indonesia. The Lancet Psychiatry. 2018;5(9):707-16.

6. Bantjes J, Kazdin AE, Cuijpers P, Breet E, Dunn-Coetzee M, Davids C, et al. A web-based group cognitive behavioral therapy intervention for symptoms of anxiety and depression among university students: Open-label, pragmatic trial. JMIR Mental Health. 2021;8(5) (no pagination).

7. Benjet C, Albor Y, Alvis-Barranco L, Contreras-Ibanez CC, Cuartas G, Cudris-Torres L, et al. Internet-Delivered Cognitive Behavior Therapy Versus Treatment as Usual for Anxiety and Depression Among Latin American University Students: A Randomized Clinical Trial. Journal of Consulting and Clinical Psychology. 2023;91(12):694-707.

8. Chen SJ, Que JY, Chan NY, Li SX, Zhang JH, Zhong Y, et al. Effectiveness of e-based cognitive behavioral therapy for insomnia on enhancing depression and insomnia outcome in Chinese youth with both diagnoses. Sleep Medicine. 2024;115(Supplement 1):171.

9. Church D, De Asis, M. A., & Brooks, A. J.,. Brief Group Intervention Using Emotional Freedom Techniques for Depression in College Students: A Randomized Controlled Trial. . Depression Research and Treatment. 2012:257172.

10. Eseadi C, Ilechukwu LC, Victor-Aigbodion V, Sewagegn AA, Amedu AN. Intervention for depression among undergraduate religious education students: A randomized controlled trial. Medicine (Baltimore). 2022;101(41):e31034.

11. Ezeudu FO, Eya NM, Nwafor SC, Ogbonna CS. Intervention for depression among chemistry education undergraduates in a Nigerian university. Journal of International Medical Research. 2020;48(1).

12. Far ST, Gharraee B, Birashk B, Habibi M. Effectiveness of acceptance and commitment therapy and cognitive therapy in patients with major depressive disorder. Iranian journal of psychiatry and behavioral sciences. 2017;11(4).

13. Fereydouni S, & Forstmeier, S.,. An Islamic Form of Logotherapy in the Treatment of Depression, Anxiety and Stress Symptoms in University Students in Iran. J Relig Health. 2022;61(1):139–57.

14. Gureje O, Oladeji BD, Montgomery AA, Araya R, Bello T, Chisholm D, et al. High- versus low-intensity interventions for perinatal depression delivered by non-specialist primary maternal care providers in Nigeria: Cluster randomised controlled trial (the EXPONATE trial). British Journal of Psychiatry. 2019;215(3):528-35.

15. Gureje O, Oladeji BD, Kola L, Bello T, Ayinde O, Faregh N, et al. Effect of intervention delivered by frontline maternal care providers to improve outcome and parenting skills among adolescents with perinatal depression in Nigeria (the RAPiD study): A cluster randomized controlled trial. Journal of Affective Disorders. 2022;312:169-76.

16. Kaaya S, Siril H, Fawzi MCS, Aloyce Z, Araya R, Kaale A, et al. A peer-facilitated psychological group intervention for perinatal women living with HIV and depression in Tanzania-Healthy Options: A cluster-randomized controlled trial. PLoS Med. 2022;19(12):e1004112.

17. Konradt CE, Cardoso TA, Mondin TC, Souza LDM, Kapczinski F, da Silva RA, et al. Impact of resilience on the improvement of depressive symptoms after cognitive therapies for depression in a sample of young adults. Trends in Psychiatry and Psychotherapy. 2018;40(3):226-31.

18. Nakku JEM, Nalwadda O, Garman E, Honikman S, Hanlon C, Kigozi F, et al. Group problem solving therapy for perinatal depression in primary health care settings in rural Uganda: an intervention cohort study. BMC Pregnancy Childbirth. 2021;21(1):584.

19. Nejati V, Fathi E, Shahidi S, Salehinejad MA. Cognitive training for modifying interpretation and attention bias in depression: Relevance to mood improvement and implications for cognitive intervention in depression. Asian J Psychiatr. 2019;39:23-8.

20. Ofoegbu TO, Asogwa U, Otu MS, Ibenegbu C, Muhammed A, Eze B. Efficacy of guided internet-assisted intervention on depression reduction among educational technology students of Nigerian universities. Medicine (Baltimore). 2020;99(6):e18774.

21. Olashore AA PS, Ogunwale A, Ita M, Tomita A, Chiliza B. The Effectiveness of Psychoeducation and Problem-solving on Depression and Treatment Adherence in Adolescents Living with HIV in Botswana: an Exploratory Clinical Trial. Child Adolesc Psychiatry Ment Health. 2023;17(1):2.

22. Osborn TL, Rodriguez M, Wasil AR, Venturo-Conerly KE, Gan J, Alemu RG, et al. Single-session digital intervention for adolescent depression, anxiety, and well-being: Outcomes of a randomized controlled trial with Kenyan adolescents. J Consult Clin Psychol. 2020;88(7):657-68.

23. Savari Y, Mohagheghi H, Petrocchi N. A preliminary investigation on the effectiveness of compassionate mind training for students with major depressive disorder: A randomized controlled trial. Mindfulness. 2021;12(5):1159-72.

24. Singla DR, MacKinnon DP, Fuhr DC, Sikander S, Rahman A, Patel V. Multiple mediation analysis of the peer-delivered Thinking Healthy Programme for perinatal depression: findings from two parallel, randomised controlled trials. British Journal of Psychiatry. 2021;218(3):143-50.

25. Srivastava P, Mehta M, Sagar R, Ambekar A. Smartteen- a computer assisted cognitive behavior therapy for Indian adolescents with depression- a pilot study. Asian J Psychiatr. 2020;50(no pagination).

26. Toth SL RF, Oshri A, Gravener-Davis J, Sturm R, Morgan- Lopez. The Efficacy of Interpersonal Psychotherapy for Depression Among Economically Disadvantaged Mothers. Dev Psychopathol. 2013;25(4 Pt 1):1065–78.

27. Xu L, Wang F, Yuan J, Wu Y, Wang X, Meng JY, et al. A randomized controlled trial of mindfulness-based cognitive therapy (MBCT) for major depressive disorder in undergraduate students in China: The efficacy, serum proinflammatory cytokines and brain-derived neurotrophic factor. Asian J Psychiatr. 2023;88(no pagination).

28. Zemestani M MS. Acceptance and Commitment Therapy for the Treatment of Depression in Persons with Physical disability: a Randomized Controlled Trial. Clin Rehabil. 2020;34(938–947).

29. Correa-Oliveira GS, L; Morais Araujo, J; A. Boin; R. Mendes Paula Pessoa; L. Rodrigues Leal and Del-Ben, C. Early Intervention for Psychosis in Emerging countries: Findings from a First-Episode Psychosis Programme in Ribeirão Preto, Brazil. European Psychiatry. 2022:S379.

30. Mlay JP, Jamieson L, Ntlantsana V, Naidu T, Bhengu BS, Paruk S, et al. Developing and testing unconditional cash transfer strategies among young adults with first-episode psychosis in South Africa: a study protocol for a pilot randomised control trial (PRS-FEP trial). BMJ Open. 2022;12(12) (no pagination).

31. Ngoc TN, Weiss B, Trung LT. Effects of the family schizophrenia psychoeducation program for individuals with recent onset schizophrenia in Viet Nam. Asian J Psychiatr. 2016;22:162-6.

32. She P, Zeng H, Yang B. Effect of self-consistency group intervention for adolescents with schizophrenia: An inpatient randomized controlled trial. Journal of Psychiatric Research. 2016;73:63-70.
